# Fish By‐Products Utilization in Food and Health: Extraction Technologies, Bioactive, and Sustainability Challenges

**DOI:** 10.1002/fsn3.71184

**Published:** 2025-11-11

**Authors:** Muhammad Waqar, Nimra Sajjad, Qudrat Ullah, S. S. Vasanthkumar, Faiyaz Ahmed, Worawan Panpipat, Rotimi E. Aluko, Lovedeep Kaur, Manat Chaijan, Temesgen Anjulo Ageru

**Affiliations:** ^1^ Food Technology and Innovation Research Center of Excellence, School of Agricultural Technology and Food Industry Walailak University Tha Sala Nakhon Si Thammarat Thailand; ^2^ Faculty of Health Deakin University, Burwood Campus Burwood Victoria Australia; ^3^ Tamil Nadu Agricultural University Coimbatore Tamil Nadu India; ^4^ Department of Basic Health Sciences, College of Applied Medical Sciences Qassim University Buraydah Saudi Arabia; ^5^ Department of Food and Human Nutritional Sciences University of Manitoba Winnipeg Manitoba Canada; ^6^ Riddet Institute, Massey University Palmerston North New Zealand; ^7^ College of Medicine and Health Sciences Wolaita Sodo University Wolaita Sodo Ethiopia

**Keywords:** bioactive peptides, circular economy, fish by‐products, functional foods, health applications, sustainable extraction

## Abstract

Fish by‐products, traditionally regarded as waste, are increasingly recognized as valuable sources of bioactive compounds, including peptides, omega‐3 fatty acids, collagen, and hydroxyapatite. These molecules exhibit significant functional properties with applications in food preservation, dietary supplementation, pharmaceuticals, and cosmeceuticals. This review explores advanced extraction technologies such as enzyme‐assisted hydrolysis, supercritical fluid extraction, and cold plasma processing, which enhance the yield and stability of bioactives while supporting zero‐waste and circular economy principles. Despite technological progress, key barriers remain, including inconsistent raw material quality, high processing costs, regulatory uncertainty, and limited industrial infrastructure. Peptides and protein hydrolysates derived from fish frames, skins, viscera, and scales have demonstrated antioxidant, antihypertensive, antimicrobial, antidiabetic activities, but translation into functional food and health products is constrained by scalability and regulatory challenges. Future work should focus on optimizing bioprocessing, validating health benefits through clinical trials, and implementing sustainable valorization frameworks. Addressing these challenges will unlock the full potential of fish by‐products in advancing food security and human health.

## Graphical abstract

Fish by‐products, often discarded, are rich sources of bioactive peptides, collagen, and omega‐3s with antioxidant, antihypertensive, and antimicrobial properties. Advanced, sustainable extraction technologies can valorize this waste into functional food, nutraceutical, and biomedical ingredients. Overcoming technical, economic, and regulatory hurdles is key to unlocking their full potential for food security and human health.

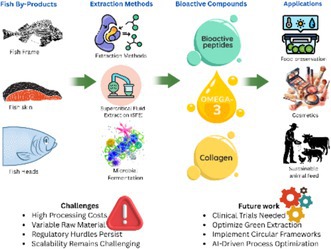

## 1 Introduction

The global demand for rich protein and functional food ingredients has driven increased interest in the sustainable utilization of marine resources, particularly fish and seafood. As the aquaculture and fisheries sectors grow to meet food security goals outlined in the United Nations' 2030 Agenda, the volume of fish processing by‐products has risen sharply (Hambrey 2017). According to the estimates of the United Nations Food and Agriculture Organization, the world food output in aquatic production is expected to amount to 202 million tons by the year 2030, as it is rise of 24 million tons of aquatic food to be consumed by humans, as compared to the 2020 levels (FAO 2022). Fish and their derivatives are important foods that supply digestible proteins, vitamins, minerals, as well as essential fatty acids that are essential in human diets in various populations.

Several parts of fish, such as heads, skin, bones, fins, scales, internal organs, and offal, are generated during fish processing. Alarmingly, over 20 billion tons of fish tissue are discarded globally each year, accounting for around 70% of fish weight (Shaw et al. 2022). In the European Union alone, fish discards amount to approximately 5.2 million tons annually (Coppola et al. 2021). These discards, estimated to comprise 20%–80% of the fish mass, are often buried or incinerated, contributing to food waste, environmental pollution, and economic inefficiency. Due to their high moisture content, fish by‐products spoil easily, necessitating immediate processing and thus affecting their potential valorization (Shaw et al. 2022). Fish have a moisture content of 50%–80%, proteins 15%–30% and fat 0%–25% of their total weight, depending on gender, age, species, gender, health condition, feed, and harvesting time (Athanasopoulou et al. 2023). These components can be extracted or hydrolyzed into functional forms, including fish protein hydrolysates (FPHs), bioactive peptides, collagen, and gelatin, all possessing significant nutritional and health benefits. Studies have linked the utilization of fish skin, head, bone, fins, and viscera and the hydrolyzed products that possess antioxidant, antihypertensive, and antibacterial properties (Halim et al. 2016; Wu et al. 2022). For example, fish waste is a significant source of collagen, which can be processed into gelatin, an ingredient that modifies the elasticity, texture, and solidity of food items (Shaw et al. 2022). In addition to food uses, fish by‐products are also critical in fishmeal and fish oil production, which accounted for 27% and 48% of global production, respectively, in 2020 (Nikoo, Regenstein, and Yasemi 2023; Wassef et al. 2023). These figures enhance the opportunities in using fishery by‐products as materials for cosmetic, pharmaceutical, and biofuel industries, among others (Lal et al. 2023).

The enhancement of bioactive components in fish can be done through processes like enzymatic hydrolysis, chemical treatment, application of ultrasound, and homogenization. Among others, enzymatic hydrolysis using proteases such as trypsin, pepsin, and alcalase is preferred due to its specificity under mild conditions that improve the quality of the hydrolysates with better functional and bioactive properties (Parvathy et al. 2020). The type of enzyme, concentration, pH, temperature, and duration have become areas of interest for researchers to obtain bioactive hydrolysates with maximum yields (Nguyen et al. 2022). Techniques such as enzyme immobilization and ultrasound‐assisted hydrolysis bring forth more efficiency and sustainability (Das and Mishra 2023).

Bioactive protein hydrolysates typically consist of 2–20 kDa peptides with antioxidant, antibacterial, antihypertensive, and immunomodulating properties (Karami and Akbari‐Adergani 2019). Hydrolysates are used in developing functional foods, supplements, and pharmaceutical formulations. FPHs and gelatin peptides improve the properties and nutritional quality of products such as food emulsions, sausages, baked products, and beverages (Nguyen et al. 2022). Moreover, these peptides support muscle protein synthesis, satiety, and cardiovascular health since they are highly digested and bioavailable (Elavarasan 2018). Beyond food application, peptides from fish by‐products have shown promise in cosmetics due to their antioxidant and wound healing properties, in the pharmaceutical industry for the management of chronic diseases such as hypertension and inflammation, as well as in agriculture as biofertilizers to enhance crop yield (Idowu et al. 2021). These multi‐purpose uses align with global sustainability trends like the EU “Blue Growth” initiative, promoting rational marine resource utilization and the advancement of the blue bioeconomy (Choudhary et al. 2021).

Despite these benefits, major challenges hinder full industrial utilization. These include the perishability and compositional variability of by‐products, limited processing infrastructure, high operational costs, and unclear regulatory frameworks. Furthermore, most bioactivity data come from in vitro studies, with limited in vivo and clinical validations available to substantiate health claims (Stevens et al. 2018). There is a need for enhancement in postharvest practice, processing technologies, in addition to favorable policies and legislation on the usage of available resources (Kimani et al. 2022).

This review aims to consolidate extraction technologies, bioactive properties, and sustainability challenges for fish by‐products, emphasizing their role in food and health applications. It critically examines the technological, environmental, and regulatory barriers that currently limit large‐scale adoption, particularly in food and health sectors, and proposes strategies and pathways to advance sustainable and value‐added utilization. By integrating a multidisciplinary team and insights, this work aims to promote comprehensive resources for researchers, industry stakeholders, and policymakers seeking to promote the effective use of marine resources in advancing functional nutrition and sustainable development.

## 2 Review Methodology

This review was conducted through a comprehensive and systematic analysis of peer‐reviewed literature focused on the valorization of fish by‐products, with an emphasis on bioactive compounds, extraction technologies, and their applications. Relevant studies were identified using major scientific databases, including ScienceDirect, PubMed, Scopus, Web of Science, and Google Scholar, employing keywords such as fish by‐products, bioactive peptides, collagen, enzymatic hydrolysis, membrane fractionation, electrodialysis with ultrafiltration, and sustainable valorization. The inclusion criteria encompassed original research and review articles published primarily between 2000 and 2025 that reported on the composition, processing, functional properties, or applications of fish‐derived by‐products, while excluding non‐English publications, non‐scientific sources, and studies lacking methodological clarity. Data were synthesized thematically into sections covering biochemical composition, advanced extraction techniques, bioactivity, challenges, and future trends, ensuring alignment with the content and structure of this manuscript.

## 3 Composition of Fish By‐Products

This section summarizes the protein, lipid, and mineral composition of fish by‐products (Table 1), highlighting their bioactive compounds and potential applications. For example, fish skins are composed of at least 35% protein, and on the other hand, viscera consist of at least 20%–30% lipids (Athanasopoulou et al. 2023). Such variations require specific extraction methods that would give high yields and bioactivity.

**TABLE 1:** Composition of fish by‐products.

By‐product	Protein content (%) (wet weight)	Lipid content (%)	Mineral content (%)	Key bioactive compounds	References
Skins	60–80	5–10	2–5	Collagen, gelatin, hyaluronic acid	Huang et al. (2016)
Bones	20–30	5–15	40–60	Hydroxyapatite, collagen	Vázquez et al. (2004)
Viscera	8–35	20–50	1–3	Enzymes, polyunsaturated fatty acids	Rubio‐Rodríguez et al. (2010)
Heads	15–25	10–20	5–10	Omega‐3 fatty acids, phospholipids	Lei et al. (2019)
Scales	30–50	1–5	30–50	Collagen, hydroxyapatite	Harikrishna et al. (2017)
Fins	10–20	2–5	30–40	Calcium carbonate, collagen	Ramakrishnan et al. (2024)
Frames	20–30	5–10	20–30	Protein hydrolysates, minerals	Yuan et al. (2024)
Guts	5–15	10–30	1–3	Probiotics, enzymes	Guérard (2007)
Liver	5–10	30–60	1–3	Omega‐3 fatty acids, vitamins	Blondeau (2016)
Ovaries	10–20	5–15	2–5	Polyunsaturated fatty acids	Ferraro et al. (2013)

As mentioned previously, the relevance of bioactive compound recovery is not only of pecuniary importance. It also plays a role in meeting the nutritional requirements of the world and helps the government in carrying out health‐related programs. For instance, fish‐derived peptides that have calcium‐binding properties reduce bone diseases such as osteoporosis, which makes it possible to consider them as one of the solutions for abating osteoporosis (He et al. 2022). Also, the antimicrobial nature of chitosan is a possible substitute for synthetic preservatives in the food packaging system (Alavi and Ciftci 2023). These applications reinstate the versatility of fishery byproducts and signify their desire to revolutionize several sectors. It is an arduous process because the composition differs from one species to another, and with different processing techniques, hence making it difficult to standardize, and the costs of operation are relatively high. In addition, given that many bioactive compounds are quite sensitive to degradation during extraction steps, there are further complications. These issues can be overwhelming, which necessitate the use of new trends and efforts from various departments for the enhancement of extraction efficiency and stability of the end products.

Literature on fish by‐product composition reveals significant variability, often attributed to species, age, diet, season, and analytical methods (e.g., AOAC vs. Kjeldahl for protein). For instance, while Athanasopoulou et al. (2023) provided broad ranges on a dry basis, earlier studies like Esteban et al. (2007), reported narrower values (49%–58% protein dry basis), highlighting the need for standardized protocols. Strengths include comprehensive proximate analyses in reviews (Coppola et al. 2021), but limitations persist in underreporting moisture adjustments, leading to incomparable data. Future research should prioritize meta‐analyses to reconcile these discrepancies and validate through multi‐species clinical trials.

### 3.1 Fish By‐Product Categorization

**FIGURE 1:**
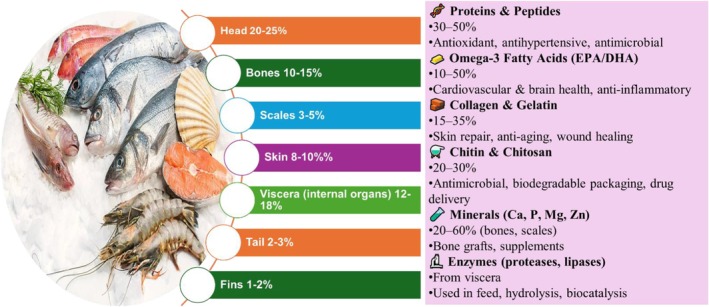
Nutritional composition and percentage distribution of fish waste parts.

Opportunities are available on how the waste materials from fish industries can be utilized and transformed into profitable products. Fish excrement is a material that is discarded even though it contains numerous proteins, lipids, and minerals. Fish by‐products, comprising 70% of fish biomass, become by‐products, including heads, skins, and viscera, with ~35% of captured fish wasted between capture and consumption (Murugan et al. 2024). Huang et al. (2016) reported 60%–80% protein in fish skins, Carpio et al. (2023) found 21%–31% in pirarucu, highlighting species‐specific variability that impacts collagen yield and extraction method selection. The fish heads, bones, skin, scales, and viscera contain collagen, peptides, enzymes, omega‐3 fatty acids, and other bioactive compounds that are thought to have usefulness in the food, medical, and industrial businesses (Coppola et al. 2021). This change of attitude from waste management to resource value creation is in line with the circular bioeconomy and environmental conservation for sustainable development. In the following context, the statistical composition, biochemical characteristics, and evidence‐based applications of valuable fish by‐products are presented to tell the story of innovation and sustainability. Figure 1 illustrates nutritional composition and percentage distribution of fish waste parts.

#### 3.1.1 Fish Skin: A Collagen‐Rich Treasure

Fish skin, as it is obtained from fish, is abundant in the markets, as rejected materials have been observed to contain proteins and collagen. On a fresh weight basis, the skin takes up 8%–10% of the fish and 4%–5% as filleting waste (Nurilmala et al. 2020) (Figure 1). The skin contains 20%–30% protein on a wet weight basis (equivalent to 60%–80% on a dry basis, assuming 60%–70% moisture), and collagen makes up 30% of the total animal protein. Similar studies on species such as meager and gilthead sea bream support these protein contents (Kandyliari et al. 2020), but Carpio et al. (2023) reported the following skin protein levels in pirarucu, 31.43% in the tail, 22.20% in the loin, and 21.92% in the belly on a wet basis, translating to approximately 70%–90% dry basis after moisture correction. Therefore, fish skin is a viable substitute for mammalian collagen without the risks associated with bovine spongiform encephalopathy (BSE). The chemical attributes of fish skin are mainly collagenous and are made up of a triple helix Glycine‐X‐Y structure with glycine, proline, and hydroxyproline making up 20%–25% of the amino acids. Hydrolysis gives rise to bioactive peptides ranging from 1 to 5 kDa with improved functionality. Gelatin, also obtained from fish skin, contains 22% monounsaturated fatty acids, including palmitic and oleic acid (Jasrotia et al. 2024), which further enhance the nutritional benefits of gelatin. The collagen derived from fish does not pose such restrictions based on religious beliefs and health concerns that may surround the use of collagen from pork or beef. Choonpicharn et al. (2015) demonstrated ACE‐inhibitory activity in tilapia skin gelatin hydrolysates, though industrial scalability remains untested, unlike broader claims in reviews (Halim et al. 2016), while Atef et al. (2021) revealed that sturgeon skin hydrolysates have antibacterial properties against Gram‐negative bacteria. Wang et al. (2016) indicated that cod skin peptides offered about a 40% decrease in cellular oxidation, which points to possible medicinal value. Fish skin is replete with collagen and gelatin, which are cheap sources in the food, cosmetic, and biomedical industries based on more than two decades of extraction studies (Nurilmala et al. 2020). In addition, every part of the fish's head contains healthy nutrients, and they make good products suitable for cognitive health enhancement. Studies on fish skin composition show inconsistencies in protein reporting; for example, Huang et al. (2016) cited 60%–80% dry basis, while Carpio et al. (2023) reported 21%–31% likely wet basis, without explicit moisture data, complicating comparisons. Variations arise from species (e.g., tropical vs. cold‐water fish), seasonal feeding, and extraction methods (acid vs. enzymatic). Strengths lie in bioactivity‐focused works (e.g., Atef et al. 2021; Wang et al. 2016), demonstrating in vitro efficacy, but limitations include lack of in vivo validation and scalability assessments (as noted in Halim et al. 2016). Gaps persist in standardizing basis reporting, with future meta‐analyses needed to address biases toward high‐yield species.

#### 3.1.2 Fish Heads

The fish head, which is normally thrown away when filleting, is rich in nutrients with a good protein content level. Constituting 10%–20% of the fish weight and 21%–25% of the filleting waste, the fish head constitutes a reasonable amount of protein that accounts for 15%–25% on a wet basis (approximately 50%–70% dry basis, based on 60%–70% moisture). According to Wangkheirakpam et al. (2019), there are various types of waste from fish, comprising heads, which contain proteins of about 58%, fat of about 19% lipids, as well as valuable minerals on a dry basis (Kandyliari et al. 2020). This by‐product, in addition to disposal, offers high‐quality polypeptides and bioactive compounds. Regarding the fish head protein content, collagen, muscle‐derived proteins, and sulfated polysaccharides (SPs) form the major constituents and contain glycine, lysine, and proline amino acids. Resources from the fish head include 60%–70% protein and other compounds such as glutathione, a powerful antioxidant peptide (Naghdi et al. 2023) on a dry basis. Lipids enriched with eicosapentaenoic acid (EPA) and docosahexaenoic acid (DHA) account for 10%–15% of the total fats in fish (Alfio et al. 2021). Tilapia head protein hydrolysate (THPH) enhances cognitive behavior in mice by reducing abnormal brain oxidative stress levels, which may be utilized against cognitive deterioration. Naghdi et al. (2023) isolated FPH from tuna heads that showed antioxidant and antibacterial properties. Zhang et al. (2021) reported salmon head hydrolysates with an IC_50_ of 0.87 mg/mL for ACE inhibition, suggesting antihypertensive potential, but clinical validation is needed. The bird's head's involvement in the creation of fish oil supports their functionality in the pharmaceutical, nutraceutical industries, and animals. In addition to this, there is a wide range of application of fish oil in animal treatment such as, Nurlatifah et al. (2024) investigated the utilization of lemuru fish oil, a valuable fish by‐product, to enhance reproductive performance in Garut ewes by improving estrous response, hormonal balance, and conception rates. Montesqrit et al. (2024) explored the use of lemuru fish oil, a fish by‐product, to improve nutrient digestibility, fiber fraction utilization, and rumen fluid fermentability in ruminants.

#### 3.1.3 Otoliths and Skeletons: The Mineral and the Protein Basis of Fish Frames

Fish frames and bones are major generators of processing waste resources that have both organic and inorganic substances. The roughly spherical head is made of cartilage with diameters of 10%–15% of the total fish weight and occupies 24%–34% of the filleting waste (Caruso et al. 2020). According to Esteban et al. (2007), fish by‐products contain protein 49.22%–57.92% while ash, 21.79%–30.16% of which bones are rich in calcium on a dry basis. The fish meal obtained from bones contains a considerable amount of minerals, in a range of 25%–35%. From the above evidence, it can be concluded that collagen comprises 30% of bone in the organic fraction, with 60%–70% being made up of calcium phosphate and hydroxyapatite (Fan et al. 2019) dry basis. Muscle‐rich frames provide amino acids such as lysine and valine, and hydrolysis increases the bioactivity of the same. Indeed, hydroxyapatite's ability to be biocompatible makes medical uses feasible, for example, in performing bone grafting (Lingam et al. 2023). Fan et al. (2019) reported the bitterness in Alaska pollock frame hydrolysates that depended on the hydrolytic enzyme, with the highest intensity observed when alkaline protease was used. Cardeira et al. (2022) used a high‐pressure method to extract the codfish frame proteins for cosmeceutical purposes. Kim et al. (2021) found that tilapia bone hydrolysates showed 68% DPPH radical scavenging at 2 mg/mL. The advantages of enzymatic hydrolysis against disposal and conformity with sustainable waste valorization are discussed by Coppola et al. (2021). Compositional data for fish frames show consistency in dry basis reporting (e.g., 49%–58% protein in Esteban et al. 2007), but variations (e.g., 26% in seabream bone per searches) stem from mineral‐rich ash content and species (e.g., bony vs. cartilaginous fish). Strengths include functional studies (e.g., Kim et al. 2021 on antioxidants), but limitations are in bitter issues (Fan et al. 2019) and lack of wet‐to‐dry conversions in older literature. Gaps include limited cross‐species comparisons; future research could use AI modeling for predictive standardization (Kurnianto et al. 2024).

#### 3.1.4 Fish Scales: Biomaterials With Unique Properties

Fish scales, though smaller in size than the major outputs, have unique characteristics that also command a good market. Scales comprising 2%–5% of the fish weight have 20%–25% protein on a wet basis (50%–70% dry basis), among which the majority contain type I collagen (Chinh et al. 2019). According to Wangkheirakpam et al. (2019), the protein fraction of scales is at 58% and the lipid fraction at 19% in fish wastes, whereas their mineral content is high enough to affirm their biomaterial value (Mo et al. 2018) dry basis.

Scales also contain type II collagen and hydroxyapatite; parts of the matrix have an arranged structure with proline and hydroxyproline occupying 20%–25% of the spectrum (Chinh et al. 2019). Essential amino acids like tryptophan enhance the nutritional value of the product, while bioactive peptides increase their functionality. They can interact with human tissues, which increases their application in biotechnology, as observed by Hussain et al. (2020). Chinh et al. (2019) extracted procollagen with a yield of 13.6% from the carp scales, which is similar to the European carp collagen. Hussain et al. (2020) designed a protein battery by utilizing Raho scales to generate power for low‐consumption devices. Biomaterials science suggested that scale‐derived collagen has 45 MPa tensile strength, which seems suitable for tissue engineering applications. Coppola et al. (2021) corroborated the position of scales as food and biomedical raw material. Scale protein content varies (20%–25% wet vs. 49%–73% dry in searches like Chinh et al. 2019), due to high ash (minerals) and species (e.g., carp vs. snapper). Literature strengths include innovative applications (Hussain et al. 2020), but are understudied compared to skins, with limitations in yield optimization and chitin focus over proteins. Gaps in seasonal/dietary influences; more comparative studies across ecosystems are needed (Mo et al. 2018).

#### 3.1.5 Fish Viscera: Enzyme and Biopeptide Hotspots

Fish viscera, accounting for as much as 12%–18% of fish weight, are also discarded as waste even though they are packed with enzymes, high levels of proteins, lipids, and polysaccharides. On a dry weight basis, they contain around 15%–20% protein (wet basis equivalent ~4%–6%, assuming 70%–80% moisture) and comprise 49.22%–57.92% of fish by‐products (Sholeh et al. 2020). Rich in digestive enzymes, pepsin, trypsin, and chymotrypsin (50%–60% of protein activity), viscera also yield lipases and aminopeptidases (Sholeh et al. 2020). Viscera hydrolysis generated a biopeptide with antioxidant and anti‐inflammatory properties (Ishak and Sarbon 2018), while the chitin of crustaceans has a positive impact on biomedical usage. Sholeh et al. (2020) isolated pepsin from tuna viscera with an activity of 101,061 AU/mg, while Riyadi et al. (2022) showed that tilapia visceral hydrolysate alleviated lung injury, and the extent of lipid peroxidation inhibition was determined to be as high as 55%. According to Vaishnav, Lal, et al. (2025) and Coppola et al. (2021), viscera have the potential attribute of generating biopeptides for use in nutraceuticals and other related products. Viscera protein reports differ (15%–20% dry in manuscript vs. 73%–78% in silages per searches), reflecting lipid dominance and processing (e.g., silage increases concentration). Strengths in enzyme‐focused studies (Sholeh et al. 2020), but limitations include contamination risks (heavy metals) and inconsistent basis reporting (Ishak and Sarbon 2018). Gaps in clinical translation of bioactivities (Riyadi et al. 2022); literature biases toward positive in vitro results, needing balanced in vivo critiques (Vaishnav, Lal, et al. 2025; Vaishnav, Mehta, et al. 2025).

## 4 Types of Bioactive Molecules

Fish by‐products are products obtained from fish, and these products have several bioactive molecules, which have different structures and functions. Peptides are generally defined as molecules of proteins that are formed by 2–20 amino acids and have their molecular weights varying from 500 to 3000 Da depending on the extraction procedure (Vaishnav, Mehta, et al. 2025). These products are either an enzymatic digest of fish protein or protein produced by microbial fermentation, and due to their unique incorporation of amino acid sequences, they exhibit high biological activity (Table 2). For example, peptides derived from tuna fish cooking juice hydrolysates contain high levels of antioxidant properties due to some hydrophobic amino acids like proline and tyrosine. Other bioactive molecules obtained from fish by‐products are lipids, especially omega‐3 polyunsaturated fatty acids (PUFAs) such as EPA and DHA. These PUFAs can increase to 50% of the total lipid content in some species, like cod liver oil. PUFAs are in the form of phospholipids, which protect them through encapsulation during digestion. Chitin, chitosan, and chitooligosaccharides are the polymers that are prevalent in such shells as shrimps and fish scales. For instance, chitosan, as a macromolecule, functions in antimicrobial and wound healing with a current market value of about $6 billion per year (Tufail et al. 2025). The major gelatin and collagen ingredients are derived from fish skins and fish bones. Collagen is made up of 70% of the skin's dry weight, and gelatin is obtained from collagen through thermal or enzymatic degradation (Nurilmala et al. 2022). Both these proteins are utilized in foods for their nutraceutical and dietary supplement purposes and applied in biomedical topics because they are biocompatible and bioactive. Some other bioactive components of fish are enzymes, carotenoids like astaxanthin, and minerals solely involved in the formation of bone tissue engineering (Han et al. 2024; Tufail et al. 2025). Literature on bioactive molecules from fish by‐products demonstrates diversity but inconsistencies in reported yields and activities; for example, peptide MW ranges 500–3000 Da vary by hydrolysis method, with enzymatic yielding higher bioactivity than microbial (e.g., antioxidant IC_50_ lower in tuna peptides) (Vaishnav, Lal, et al. 2025; Vaishnav, Mehta, et al. 2025). Strengths include market‐oriented reviews (Tufail et al. 2025 on chitosan), but limitations lie in overreliance on in vitro assays without addressing bioavailability or species‐specific differences (Nurilmala et al. 2022). Gaps include limited clinical trials for health claims (Han et al. 2024) and potential biases toward positive outcomes; future studies should integrate multi‐omics for structure–function validation and compare with synthetic alternatives for sustainability.

**TABLE 2:** Bioactive properties and applications of fish species in functional foods and health products.

Fish species	Raw material	Key bioactive properties	Applications	References
Cod (not specified)	Frame	Exhibits antioxidant and ACE inhibitory effects	Antioxidant and antihypertensive food ingredient	Jeon et al. (1999)
*Theragra chalcogramma*	Skin	Inhibits ACE (e.g., peptides Gly‐Pro‐Leu, Gly‐Pro‐Met)	Blood pressure management	Byun and Kim (2001)
Pollack (not specified)	Skin	Shows antioxidant effects (e.g., peptides with hydroxyproline)	Antioxidant supplement	Kim et al. (2001)
Yellowtail (not specified)	Bone	Shows antioxidant and ACE inhibitory effects	Antioxidant and antihypertensive ingredient	Morimura et al. (2002)
Yellowtail (not specified)	Scale	Displays antioxidant and ACE inhibitory effects	Antioxidant and antihypertensive agent	Ohba et al. (2003)
Yellowtail (not specified)	Bone	Displays antioxidant and ACE inhibitory effects	Antioxidant and antihypertensive agent	Ohba et al. (2003)
*Limanda aspera*	Frame	Displays antioxidant effects (e.g., peptide N‐terminal RPDFDLEPPY)	Antioxidant supplement	Jun et al. (2004)
Sea bream (not specified)	Scale	Reduces blood pressure (e.g., peptides GY, VY, GF, VIY)	Antihypertensive supplement	Fahmi et al. (2004)
*Limanda aspera*	Frame	Prevents blood clotting	Anticoagulant agent	Rajapakse et al. (2005)
*Theragra chalcogramma*	Frame	Shows antioxidant effects (e.g., peptide Leu‐Pro‐His‐Ser‐Gly‐Tyr)	Antioxidant food additive	Je et al. (2005)
*Theragra chalcogramma*	Frame	Inhibits ACE (e.g., peptide Phe‐Gly‐Ala‐Ser‐Thr‐Arg‐Gly‐Ala)	Blood pressure control	Je et al. (2005)
*Johnius belengerii*	Skin	Shows antioxidant effects (e.g., peptide HGPLGPL)	Antioxidant ingredient	Mendis et al. (2005)
*Johnius belengerii*	Bone	Binds calcium	Calcium‐binding supplement	Jung et al. (2005)
*Johnius belengerii*	Frame	Exhibits antioxidant effects (e.g., peptide GSTVPERTHPACPDFN)	Antioxidant ingredient	Kim et al. (2007)
*Johnius belengerii*	Frame	Binds calcium (e.g., peptide VLSGGTTMYASLYAE)	Calcium‐binding supplement	Jung and Kim (2007)
Tuna (not specified)	Bone	Shows antioxidant effects (e.g., peptide VKAGFAWTANQQLS)	Antioxidant supplement	Je et al. (2007)
Shark (not specified)	Not specified	Reduces blood pressure (e.g., peptides Cys‐Phe, Glu‐Tyr)	Antihypertensive supplement	Wu et al. (2008)
*Clupea harengus*	Bone	Reduces blood pressure (e.g., peptides Leu‐Ser‐Gly‐Phe‐Asp‐Thr)	Antihypertensive supplement	Lindqvist et al. (2008)
*Gadus morhua*	Bone	Exhibits antioxidant effects	Antioxidant food additive	Šližytė et al. (2009)
*Oreochromis* sp.	Collagen	Enhances facial skin quality	Cosmeceutical for skin care	Chai et al. (2010)
*Lutjanus vitta*	Skin	Exhibits antioxidant effects	Antioxidant food additive	Khantaphant (2010)
*Gadus macrocephalus*	Gelatin	Shows antioxidant and ACE inhibitory effects (e.g., peptides Thr‐Cys‐Ser‐Pro, Thr‐Gly‐Gly‐Gly‐Asn‐Val)	Antioxidant and antihypertensive food ingredient	Ngo et al. (2011)
*Thunnus tonggol*	Dark muscle	Inhibits breast cancer cell growth (e.g., peptides Leu‐Pro‐His‐Val‐Leu‐Thr‐Pro‐Glu‐Ala‐Gly‐Ala‐Thr)	Anticancer supplement	Hsu et al. (2011)
*Parastromateus niger*	Viscera	Exhibits antioxidant effects (e.g., peptide Ala‐Met‐Thr‐Gly‐Leu‐Glu‐Ala)	Antioxidant health product	Jai Ganesh et al. (2011)
*Magalaspis cordyla*	Viscera	Shows antioxidant effects (e.g., peptide Ala‐Cys‐Phe‐Leu)	Antioxidant ingredient	Kumar et al. (2011)
*Misgurnus anguillicaudatus*	Not specified	Inhibits ACE	Blood pressure regulation	Li et al. (2012)
*Oreochromis niloticus*	Skin	Displays antioxidant effects (e.g., peptides Glu‐Gly‐Leu, Tyr‐Gly‐Asp‐Glu‐Tyr)	Antioxidant supplement	Zhang et al. (2012)
*Paralichthys olivaceus*	Not specified	Shows antioxidant effects (e.g., peptides VCSV, CAAP)	Antioxidant food additive	Ko et al. (2013)
*Oncorhynchus keta*	Skin	Lowers blood pressure (e.g., peptide Gly‐Leu‐Pro‐Leu‐Asn‐Leu‐Pro)	Antihypertensive agent	Lee et al. (2014)
*Acipenser schrenckii*	Skin	Exhibits antioxidant and cryoprotective effects (e.g., peptide Pro‐Ala‐Gly‐Tyr)	Antioxidant and cryoprotectant ingredient	Nikoo et al. (2014)
*Saurida elongata*	Not specified	Reduces blood pressure (e.g., peptide Arg‐Val‐Cys‐Leu‐Pro)	Antihypertensive functional ingredient	Wu et al. (2015)
*Navodon septentrionalis*	Skin	Shows antioxidant effects (e.g., peptides Gly‐Ser‐Gly‐Gly‐Leu, Gly‐Pro‐Gly‐Gly‐Phe‐Ile)	Antioxidant food additive	Chi, Wang, Hu, et al. (2015)
*Chanos chanos*	Collagen	Binds iron	Iron‐binding supplement	Huang et al. (2015)
*Navodon septentrionalis*	Head	Exhibits antioxidant effects (e.g., peptides Trp‐Glu‐Gly‐Pro‐Lys, Gly‐Pro‐Pro)	Antioxidant supplement	Chi, Wang, Wang, et al. (2015)
*Paralichthys olivaceus*	Not specified	Inhibits ACE (e.g., peptides MEVFVP, VSQLTR)	Blood pressure management	Ko et al. (2016)
*Benthosema pterotum*	Not specified	Protects nerve cells (e.g., peptides Phe‐Tyr‐Tyr, Asp‐Trp)	Neuroprotective supplement	Chai et al. (2016)
*Raja porosa*	Cartilage	Shows antioxidant effects (e.g., peptides Phe‐Ile‐Met‐Gly‐Pro‐Tyr, Gly‐Pro‐Ala‐Gly‐Asp‐Tyr)	Antioxidant food additive	Pan et al. (2016)
*Meuchenia* sp.	Not specified	Lowers blood pressure	Antihypertensive agent	Salampessy et al. (2017)
*Oncorhynchus keta*	Skin	Improves brain function	Neurobehavioral supplement	Xu et al. (2020)
*Oncorhynchus keta*	Collagen	Supports learning, memory, and bone development (rich in amino acids like Gly, Pro, Asp)	Brain and bone health supplement	Xu et al. (2020)
*Oncorhynchus keta*	Pectoral fin	Displays antioxidant effects (e.g., peptide Phe‐Leu‐Asn‐Glu‐Phe‐Leu‐His‐Val)	Antioxidant ingredient	Xu et al. (2020)
*Oreochromis niloticus*	Viscera/carcass	Shows antioxidant effects	Antioxidant food additive	Silva et al. (2021)
Tuna (not specified)	Frame	Lowers blood pressure (e.g., peptide Gly‐Asp‐Leu‐Gly‐Lys‐Thr‐Thr‐Thr‐Val‐Ser‐Asn‐Trp‐Ser‐Pro‐Pro‐Lys‐Try‐Lys‐Asp‐Thr‐Pro)	Antihypertensive health product	Lee et al. (2022)
*Oncorhynchus keta*	Viscera	Reduces blood pressure (e.g., peptides Leu‐Leu‐Tyr‐Leu‐Asn)	Antihypertensive ingredient	Jang et al. (2022)
*Oncorhynchus keta*	Viscera	Exhibits antioxidant and ACE inhibitory effects	Antioxidant and antihypertensive food ingredient	Jang et al. (2022)
*Exocoetus volitans*	Bone	Exhibits antioxidant and antiproliferative effects	Antioxidant and anticancer supplement	Nazeer et al. (2023)

## 5 Extraction Technologies

### 5.1 Conventional Methods

#### 5.1.1 Enzymatic Hydrolysis

Enzymatic hydrolysis is another widely used method for generating FPHs, as it employs the use of specific proteolytic enzymes that yield better results in the breakdown of fish proteins into peptides and free amino acids. This is a process that is carried out by enzymes that gently split the peptide bonds using limited heat, pressure, and pH level, among other features, which preserve the newly formed peptides. The process begins with the accumulation of fishery leftovers like frame, trimmings, visceral, etc., which are treated enzymatically using proteolytic enzymes like trypsin, pepsin, alcalase, etc. The specificity of these enzymes makes it possible to achieve selective hydrolysis for the release of peptides with the required bioactive characteristics.

Studies have thoroughly documented this method's effectiveness, hence proving its practicality. Wisuthiphaet et al. (2015) showed the potential of enzymatic hydrolysis to reduce proteins into functional or bioactive peptides. Parvathy et al. (2018) reported that the hydrolysis process uses a low amount of enzyme under mild conditions, which does not lead to amino acid degradation. Rubio‐Rodríguez et al. (2010) obtained improved protein recovery and bioactive compounds extraction, as well as investigated the various enzyme sources such as plant (papain, bromelain), animal (trypsin, chymotrypsin), and microbial (alcalase, flavourzyme). Enzymatic hydrolysis is preferred since it is accurate and mild in its operation. The amount of enzyme required as a catalyst remains very small, as it can be inactivated after the reaction, and the process generates a minimal amount of waste products (Parvathy et al. 2018). This prevents the degradation of amino acids, which contributes to the production of high‐quality hydrolysates with desirable nutritional and bioactive properties. One major advantage of this method is the freedom to choose the enzyme to use and the conditions under which the peptide will be broken down, making it possible to incorporate the product in food and nutraceuticals. As for the limitations, enzymatic hydrolysis has few. Getting high‐quality enzymes is expensive, and this is even more so when one attempts to generate large quantities of hydrolysates. Furthermore, due to the physical and chemical properties involved in the process, control of reaction parameters such as pH, reaction duration, and temperature poses a challenge in process scale‐up and makes the process more operationally intensive. There is also general enzyme specificity that determines the concentration of peptides made based on the substrate and protease used. This compositional data informs the selection of appropriate extraction methods to maximize recovery efficiency and functional value (Table 3). Enzymatic hydrolysis yields higher bioactivity than chemical methods but faces scalability challenges (Das and Mishra 2023), necessitating further optimization for industrial applications.

**TABLE 3:** Enzyme‐assisted extraction and bioactive properties of fish‐derived compounds.

Raw material	Enzyme/method	Bioactive property	Application	References
Squid mantle	Alcalase	Nutritional protein hydrolysate	Infant nutrition	Córdova‐Murueta and García‐Carreño (2002)
Cod skin	Papain	Collagen hydrolysate with antioxidant activity	Skin health, anti‐aging	Mendis et al. (2005)
Rainbow trout	Ultra‐filtered hydrolysate	Enhanced growth rate and feed efficiency	Sustainable fish meal alternative in aquaculture	Aksnes et al. (2006)
Catfish frame protein	Flavourzyme	Enhanced feed efficiency and growth	Aquafeed additive	Kristinsson (2007)
Shark cartilage	Enzymatic hydrolysis	Chondroitin sulfate extraction	Joint health supplement	Sim et al. (2007)
Marine by‐products (general)	Various enzymes	Comprehensive review of enzymatic utilization	Overview of marine waste valorization	Ferraro et al. (2010)
Tuna viscera	Enzymatic hydrolysis	Antioxidant and antihypertensive peptides	Functional ingredients	Samaranayaka and Li‐Chan (2011)
Tuna cooking juice	Enzymatic hydrolysis	Antioxidant and antihypertensive	Nutraceuticals	Bougatef et al. (2012)
Alaska pollock frame	Alcalase	Antioxidant peptides	Value addition to fish waste	Hou et al. (2012)
Mollusk meat waste	Enzymatic digestion	Peptides for antimicrobial use	Biopreservatives	Harnedy and FitzGerald (2012)
Anchovy waste	Neutrase	Protein hydrolysate with foaming capacity	Food industry	Ghaly et al. (2013)
Shrimp waste	Alcalase	Cryoprotective (stable surimi storage)	Alternative cryoprotectant for surimi	Dey and Dora (2014)
Unicorn leather jacket skin	Glycyl endopeptidase (papaya latex)	Cryoprotective, delays lipid oxidation	Prevents protein denaturation in mackerel mince	Karnjanapratum and Benjakul (2015)
Herring viscera	Pepsin	Protein hydrolysates with antioxidant activity	Food additives	Bao et al. (2017)
Tuna frame waste	Alcalase	Functional peptides with good emulsifying properties	Food emulsifier	Sayana and Sirajudheen (2017)
Squid waste	Acid hydrolysis	Protein hydrolysate with high nitrogen content	Animal feed	Martin Xavier et al. (2017)
Cod fish mince	Protamex, flavourzyme	Cryoprotective (stable after freeze–thaw)	Alternative cryoprotectant for frozen fish	Jenkelunas and Li‐Chan (2018)
Dried squid head	Flavourzyme	Sweet‐umami flavor	Flavored functional food ingredient	Sukkhown et al. (2018)
*Cyclina sinensis*	Trypsin, neutral protease	Inhibits cancer cell growth (A549, Hela, HepG2)	Potential anticancer agent	Xu et al. (2018)
Yellow stripe trevally waste	Alcalase, flavourzyme	Antioxidant, iron chelation	Functional food ingredient	Klompong et al. (2008)
Fish roe	Trypsin	Hydrolysate with high lysine content	Baby food	Rajabzadeh et al. (2018)
Milkfish collagen	*B. thuringiensis* , *B. licheniformis*	Antifungal ( *Candida albicans* ), antioxidant	Not specified	Kusumaningtyas et al. (2019)
Silver carp byproducts	Protamex, alcalase	Cryoprotective (surimi, 6 freeze–thaw cycles)	Cryoprotectant for surimi (low sweetness)	Zhou et al. (2019)
Skipjack tuna skin	Papain	Collagen peptides, antioxidant	Skincare and nutraceuticals	Lin, Chen, Jin, et al. (2019); Lin, Chen, Tsai, and Chen (2019)
Rainbow trout viscera	Alcalase	Antimicrobial peptides (e.g., against *E. coli* )	Natural preservative	Naseri et al. (2020)
Anchovy waste	Alcalase, papain	Bioactive peptides with DPPH scavenging activity	Value‐added functional food	Giannetto et al. (2020)
Mackerel hydrolysates	Enzymatic hydrolysis	Immunomodulatory peptides	Health supplements	Korczek et al. (2020)
Crab shell waste	Enzymatic hydrolysis	Chitin and protein recovery	Biomedical and agricultural use	Ding et al. (2020)
Sardine heads	Autolysis	Peptide‐rich broth with nutritional potential	Soup base	Sajib (2021)
Keropok Lekor byproducts	Fermentation ( *Lactobacillus casei* )	Antimicrobial, antioxidant	Inhibits *Salmonella*, *E. coli* , and *Listeria* growth	Abd Rashid et al. (2022)
Cod head	Ultrasound pretreatment	Increased soluble peptide content	Enhanced enzymatic hydrolysis of fish byproducts	Wang et al. (2022)
Fish processing byproducts	Amylase, transaminase	Improved growth and protein efficiency in fish	Dietary supplement for striped murrel fingerlings	Siddaiah et al. (2022)
Salmon skin	Pepsin	Antimicrobial peptides	Biopreservation	Zhang et al. (2022)
Fish muscle (various species)	Pepsin and trypsin	Production of peptides with improved digestibility	Clinical nutrition	Patil et al. (2022)
Cod skin	Fermentation	Gelatin extraction with bioactive potential	Nutraceuticals	Cardeira et al. (2022)
Hake viscera	Pepsin hydrolysis	High protein digestibility and amino acid content	Dietary supplement	Patil et al. (2022)
Bighead carp heads, Rainbow trout viscera	Alcalase	Antimicrobial, antioxidant	Active packaging development	Naghdi et al. (2023)
Tilapia skin collagen	Alcalase	Cryoprotective (antifreeze peptides)	Cryoprotectant for frozen scallop muscles	Cao et al. (2023)
White shrimp heads	Ultrasonic‐assisted extraction	High extraction yield, umami enhancement	Shrimp flavor concentrate development	Duppeti et al. (2023)
Bighead carp waste	Trypsin	Angiotensin‐converting enzyme (ACE) inhibition	Antihypertensive agent	Gao et al. (2023)
Fish frame (misc.)	Alcalase	Hydrolysates with antioxidant and emulsifying properties	Functional foods	Naghdi et al. (2023)
Swordfish head muscle	Alcalase, savinase	Antioxidant activity	Functional ingredients	Elgaoud et al. (2024)
Skipjack tuna viscera	Alcalase, pancreatin	DPPH scavenging, iron chelating, nitric oxide inhibition	Health supplements, functional products	Mahoonak et al. (2024)
Sturgeon bone	Trypsin	Antioxidant peptides (< 3 kDa)	Functional foods, pharmaceuticals	Zhang et al. (2024)
Prawn and shrimp waste	Flavourzyme, protamex	Antioxidant, antimicrobial, nutritional enhancement	Functional food emulsions with furcellaran/astaxanthin	Tkaczewska et al. (2024)
Rainbow trout waste	Alcalase ( *Bacillus licheniformis* )	Antibacterial ( *Pseudomonas aeruginosa* )	Toxin prevention, perishable goods safety	Safari (2024)
Shrimp head	Alcalase	Flavor enhancement (savory, umami)	Substitute for commercial flavor enhancers	Yuniarti et al. (2024)
Shrimp heads, cephalothoraxes	Not specified	High protein, antioxidant, improved digestibility	Protein source in dog food formulations	Guilherme‐Fernandes et al. (2024)
Horse mackerel muscle	Trypsin	Bioactive peptides with lipid‐lowering effect	Cardiovascular health	Martínez et al. (2024)
Sardine heads and viscera	Alcalase	DPPH radical scavenging	Functional supplements	Manni et al. (2024)
Cod frame	Papain	Bioactive peptides with ACE‐inhibitory property	Cardiovascular health	Zalewski et al. (2024)
Mackerel muscle	Protamex	Peptides with anti‐inflammatory properties	Functional food ingredient	Lim et al. (2024)
Tuna viscera	Fermentation	Flavor‐enhancing hydrolysate	Condiment production	Tian et al. (2024)
Tilapia frame	Alcalase	Protein hydrolysates with antioxidant capacity	Functional ingredients in food	Huang et al. (2025)
Sardine muscle	Trypsin, alcalase	ACE‐inhibitory and antioxidant	Functional food	Calado et al. (2025)
Anchovy waste	Protease from *Bacillus* sp.	High degree of hydrolysis, functional peptides	Protein supplement	Tang et al. (2025)
Tuna waste	Trypsin	Fish protein hydrolysate as fertilizer	Agricultural input	Prajaputra et al. (2025)
Shrimp waste	Trypsin	Chitin recovery and protein hydrolysate	Waste valorization	Eggink et al. (2025)
Salmon by‐products	Endopeptidase	Hydrolysate with iron‐chelating ability	Mineral supplementation	Watanabe et al. (2025)
Grass carp	Alcalase	Peptide‐zingerone complex used to check in vitro antiproliferative effects	Functional food ingredient	Huang et al. (2024)

#### 5.1.2 Microbial Fermentation

Microbial fermentation uses microorganisms, whereby enzymes known as proteases break down fish proteins existing outside the cell into smaller peptides as well as amino acids. It involves using microbes like *Aspergillus oryzae* or *Saccharomyces cerevisiae* on fishery waste and permitting the microbes to secrete enzymes that hydrolyze proteins. It can be done in both solid and liquid phases, although the use of the solid phase is carried out under low moisture to ease the later drying operations of the product. The resulting peptides can be used intracellularly without extraction. Microbial fermentation is hence highlighted by Xu et al. (2023) as an economical procedure that helps in the breakdown of proteins into peptides. The benefits of this method include short drying time, while Abuine et al. (2019) explored particular microorganisms such as *Aspergillus oryzae* and *Streptococcus thermophilus* , which proved their ability to perform hydrolysis. According to Raveschot et al. (2018), optimizing the hydrolysis method offered the chance to inactivate antinutritional and allergenic compounds, and hence improve the safety of the hydrolysate. The fermentation technique makes the process cheaper when compared to the use of purified enzymes since it involves exploiting the natural biological processes that are already in existence (Xu et al. 2023). It seems that relative to the liquid type of fermentation, solid‐state fermentation is easier in the downstream processing since it contains lower moisture, hence reducing the cost of drying. On the same note, it can enhance product purity by degrading undesirable compounds (Raveschot et al. 2018) and is appropriate for the food industry. Microbial fermentation has the following disadvantages: The process is influenced by environmental factors, hence non‐fixable for a certain yield (Xu et al. 2023). Such factors lessen the chances of microbial strains that increase the complexity of purification and are unsafe (Xu et al. 2023). Moreover, it is expensive in terms of cost to optimize and upscale the process because it is sensitive to fermentation conditions and microbial control (Raveschot et al. 2018).

#### 5.1.3 Acidic Hydrolysis

Acidic hydrolysis exposes fish proteins to strong acids, particularly hydrochloric and sulfuric acids, which facilitate the cleavage of fish proteins into peptides and amino acids. This is a chemical process carried out at high temperatures and pressure, in that it breaks down proteins completely into a solution that is then made neutral, after which it is pasted or powdered. The drastic conditions guarantee total protein hydrolysis and account for its effectiveness in the valorization of fish byproducts. Siddik et al. (2021) added it to the list of widely used and efficient methods of FPH production, attributed to its capability of deproteinizing fishery waste. An acid‐soluble method is effective in the sense that it achieves complete hydrolysis of the protein component of waste products (Siddik et al. 2021). It is easier and more efficient than the enzymatic methods and does not involve the use of complex and expensive equipment (Petrova et al. 2018). The process is also highly efficient, especially with relatively shorter throughput times. The acidic solutions used in this method operate under severe conditions, which break down nourishing amino acids and thus diminish the nutritional value of generated protein hydrolysates (Elavarasan 2018). In addition, researchers find it difficult to control chemical reactions, which results in inconsistent characteristics of peptides from different batches of hydrolysis (Petrova et al. 2018). The process of neutralizing the acidic solution demands additional time and produces waste, which creates environmental worries.

#### 5.1.4 Alkaline Hydrolysis

Alkaline hydrolysis uses alkali agents from the group of sodium hydroxide, calcium hydroxide, and potassium hydroxide to break fish proteins into water‐soluble peptides. Osmotic pressure at temperatures between 27°C and 54°C transforms fish protein concentrates into water‐soluble peptides during this chemical process. The process aims to hydrolyze proteins under basic conditions, often targeting specific amino acids like tryptophan, which remain stable in alkaline environments. Elavarasan (2018) described the use of alkali to produce soluble peptides, while Pasupuleti and Braun (2010) detailed the temperature range and a commonly used agent like sodium hydroxide. Çevikkalp et al. (2016) highlighted its utility in detecting tryptophan and the chemical reactions involved, such as racemization and β‐elimination, which occur under alkaline conditions. Alkaline hydrolysis operates at relatively low temperatures, reducing energy costs compared to acidic hydrolysis (Pasupuleti and Braun 2010). The conservation of tryptophan under alkaline conditions gives alkaline hydrolysis a unique value for nutritional product development (Çevikkalp et al. 2016). With simple operations and quick procurement of alkali agents, the industrial application of this method becomes more convenient, but sodium hydroxide consumption may decrease both functional properties and nutritional content (Elavarasan 2018). The reaction method causes two major issues, which include L‐amino acid conversion into D‐amino acids and toxic compound formation through β‐elimination to produce lysinoalanine. The suitability of fish protein hydrolysate for food‐grade products and product safety is restricted by these factors. Enzymatic hydrolysis produces high‐quality products, yet features difficulty in scalability at a reasonable cost. Acidic hydrolysis delivers both operational ease and processing efficiency at the expense of nutrient depleting effects on the system and detrimental environmental effects. When alkaline hydrolysis occurs, it protects the amino acid content while introducing risks of toxicity along with functional degradation. Fishery waste valorization shows promise through these various methods while scientists continue investigating better implementations in food and non‐food industries.

### 5.2 Novel Methods

#### 5.2.1 Ultrasound‐Assisted Extraction (UAE)

Ultrasound‐assisted hydrolysis enhances yield and sustainability (Liu et al. 2024). Ultrasound‐assisted extraction (UAE) operates through the generation of high‐frequency compression waves (≥ 20 kHz) in liquid media, which induce cavitation. The implosion of cavitation bubbles creates localized zones of high pressure and temperature, effectively disrupting cellular matrices in fish by‐products to release proteins such as collagen and peptides. Chen et al. (2023) demonstrated that UAE significantly enhanced collagen extraction from tilapia scales by optimizing temperature, duration, and ultrasound power, achieving nearly double the yield compared to conventional acid extraction. The increased efficiency was attributed to enhanced matrix disruption. Similarly, Li, Tian, et al. (2022) reported the successful application of UAE in isolating antioxidant peptides from tuna skin, highlighting improved peptide bond cleavage, solubility, and bioactivity due to effective cavitation. UAE offers advantages such as increased yield, improved mass transfer, and reduced extraction time. However, its high energy requirements can lead to elevated operational costs. Furthermore, improper control of ultrasound parameters may result in protein denaturation, negatively impacting the functional properties of the extracted peptides.

#### 5.2.2 High‐Pressure Processing (HPP)

High‐pressure processing (HPP) involves subjecting fish by‐products to pressures ranging from 100 to 600 MPa, leading to the disruption of hydrogen and hydrophobic bonds in protein structures. This process enhances protein solubility and limits the need for chemical agents, thereby preserving protein integrity. Cardeira et al. (2022) employed HPP in combination with supercritical CO_2_ and subcritical water to extract protein compounds from *Gadus morhua* (codfish) frames. The study confirmed HPP's effectiveness in producing high‐value proteins suitable for cosmeceutical applications. Structural modifications induced by pressure can lead to enhanced extraction efficiency and bioactive potential. HPP aligns well with sustainable processing objectives by reducing chemical inputs and increasing the valorization of by‐products. However, the high cost of HPP equipment poses a significant barrier to small‐scale adoption. Additionally, careful optimization of pressure parameters is essential, as excessive pressure may impair protein functionality.

#### 5.2.3 Pulsed Electric Field (PEF)

Pulsed Electric Field (PEF) technology utilizes short, high‐voltage electric pulses (10–80 kV/cm) to induce electroporation in the cell membranes of fish by‐products. This process increases membrane permeability, facilitating the release of intracellular compounds such as collagen and enzymes without causing significant structural degradation. Li and Zhu (2024) reported that PEF‐assisted collagen extraction from fish skin yielded higher recoveries due to enhanced membrane permeability. The non‐thermal nature of PEF preserves protein structural integrity and functional bioactivity. The process offers rapid extraction rates and minimal denaturation; however, its high initial capital cost remains a key limitation. Additionally, its efficiency is tissue‐dependent, with dense or fibrous materials requiring higher energy inputs and additional pretreatment, which limits scalability and cost‐effectiveness.

#### 5.2.4 Microwave‐Assisted Extraction (MAE)

**FIGURE 2:**
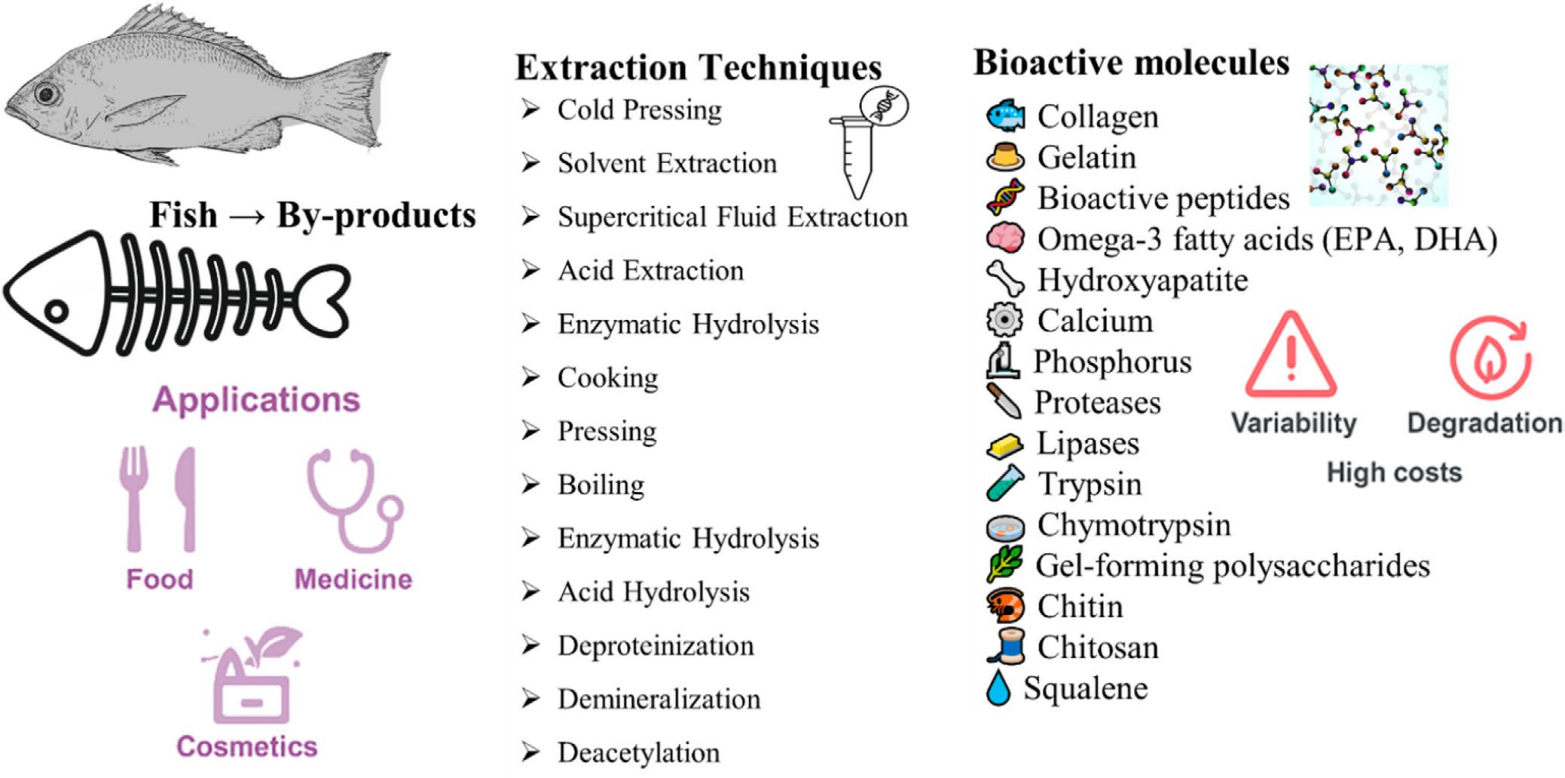
Fish by‐products: extraction, bioactive properties, applications, and challenges.

Microwave‐assisted extraction (MAE) employs electromagnetic radiation (typically at 2.45 GHz) to rapidly heat fish by‐product mixtures. The process induces cellular disruption through thermal expansion and vaporization, thereby enhancing protein release, particularly collagen and peptide fractions. Nguyen et al. (2022) demonstrated the effectiveness of MAE in extracting gelatin from fish bones, reporting significantly increased yields and reduced extraction time. Rapid matrix degradation facilitated efficient recovery, attributed to effective thermal energy transfer. However, improper temperature control can lead to thermal degradation of heat‐sensitive proteins. Moreover, MAE requires specialized equipment, and its performance may be compromised by heterogeneous material composition and uneven heat distribution. Figure 2 illustrates the extraction techniques, bioactive compounds derived from fish by‐products, and their applications in food, medicine, and cosmetics, along with associated challenges.

### 5.3 Peptide Separation Techniques

To enable the commercial use of bioactive peptides, efficient purification from complex hydrolysate mixtures is essential. Membrane filtration technologies such as ultrafiltration (UF), nanofiltration (NF), electrodialysis with ultrafiltration (EDUF), and expanded membrane reactor (EMR) systems are increasingly employed due to their scalability and high throughput compared to chromatographic techniques.

#### 5.3.1 Membrane Filtration

Membrane filtration relies on selective permeability, enabling separation based on molecular size, shape, or charge. These processes are driven by pressure, electric fields, or concentration gradients. Pressure‐driven methods such as microfiltration (MF), UF, NF, and reverse osmosis (RO) facilitate size‐based exclusion, with the molecular weight cut‐off (MWCO) indicating the smallest solute retained at 90% efficiency. García‐Depraect et al. (2021) emphasized that transmembrane pressure (TMP) governs flux rates, though excessive pressure can cause concentration polarization (CP) and membrane fouling, reducing performance. CP forms a solute concentration gradient near the membrane surface, decreasing flux rate (Davies et al. 2011). TMP optimization is, therefore, critical for balancing flux and selectivity, minimizing fouling. Selectivity is quantified by the sieving coefficient, influenced by solute and membrane properties.

#### 5.3.2 Ultrafiltration (UF) for Bioactive Peptide Recovery

Ultrafiltration employs membrane cut‐offs to fractionate peptides by molecular size. Larger molecules are retained, while smaller peptides pass through the membrane under pressure‐driven flow. A 4 kDa modified PES (m‐PES) membrane outperformed PES and PS in recovering < 2 kDa antioxidant peptides from *Saithe* fish protein hydrolysates (Chabeaud et al. 2009). Furthermore, increasing feed concentration (30–150 g/L) improved medium peptide (0.5–3 kDa) recovery, while higher TMP (10–30 bar) enhanced the extraction of small peptides (< 1 kDa) due to varied CP dynamics. A 50 kDa ceramic UF membrane extracted antioxidant peptides from egg yolk hydrolysates with > 90% efficiency, outperforming regenerated cellulose membranes (Beaubier et al. 2021). Previous works have reported that a 1 kDa membrane yielded 70.3% pure neokyotorphin (653 Da) from bovine hemoglobin hydrolysate, but at lower productivity (0.5 g m^−2^ h^−1^) than a 3 kDa membrane (9.8 g m^−2^ h^−1^), highlighting the trade‐off between purity and flux (Alavi and Ciftci 2023; Boukil et al. 2018).

#### 5.3.3 Enzymatic Membrane Bioreactor (EMR) for Simultaneous Hydrolysis and Separation

Enzymatic Membrane Bioreactors (EMRs) offer a dual‐function platform enabling the simultaneous enzymatic hydrolysis and separation of peptides. These systems retain both substrates and enzymes in the retentate while continuously collecting peptides through the permeate (Sitanggang et al. 2022). Among the common EMR configurations, free‐enzyme systems where enzymes accumulate on the membrane surface are more prevalent due to their cost‐effectiveness compared to biocatalytic EMRs, in which enzymes are immobilized on the membrane matrix (Beaubier et al. 2021).

A 10 kDa ultrafiltration (UF) membrane reduced casein phosphopeptide (CPP) yield compared to 1 kDa and 5 kDa membranes, even though the < 2 kDa peptides constituted most of the product. Despite this, 1 and 5 kDa membranes enabled the isolation of peptides with demonstrated higher angiotensin‐converting enzyme (ACE) inhibitory activity, while the 10 kDa membranes achieved the highest productivity (3.2 g L^−1^ h^−1^) for casein peptides (Eisele et al. 2013). Employing a 3 kDa EMR with continuous water addition enhanced protein conversion by 62.7% and increased antihypertensive peptide output from *Porphyra yezoensis* by 2.6‐fold with substrate addition. A 55.3% protein conversion rate using a 5 kDa EMR for wheat germ peptide production outperformed traditional methods by 36.17%. Ceramic EMRs (10 kDa) showed superior performance for wheat gluten and casein hydrolysates (Berends et al. 2016). A 79% protein conversion efficiency was achieved for corn germ peptides using a continuous 1 kDa EMR, while an 81.9% conversion rate produced peptides with blood‐pressure‐lowering effects in rats using a 5 kDa EMR and gradient substrate feeding (Cui et al. 2011).

#### 5.3.4 Nanofiltration (NF) for Charge‐Based Separation of Bioactive Peptides

Nanofiltration (NF) membranes integrate both size‐ and charge‐based separation mechanisms, leveraging the Donnan effect in polyamide membranes to preferentially permeate positively charged peptides while retaining negatively charged species (Alavi and Ciftci 2023). Lapointe et al. (2014) showed that at pH 5, acidic peptides (pI < 5) passed more readily through a 2.5 kDa NF membrane, while at pH 9, neutral and basic peptides showed increased transmission. Demers‐Mathieu et al. (2013) successfully recovered antibacterial whey hydrolysates with 2.5 kDa NF membranes. While Peptide purification from fish by‐products, such as salmon skin hydrolysates, yields ACE‐inhibitory peptides (Munawaroh et al. 2024). Yu et al. (2020) designed positively charged NF membranes that improved the separation of basic peptides from neutral and acidic peptides via enhanced charge discrimination.

#### 5.3.5 Electrodialysis With Ultrafiltration Membranes (EDUF) for Targeted Recovery of Bioactive Peptides

Electrodialysis with ultrafiltration (EDUF) combines electrokinetic migration with ultrafiltration to selectively recover peptides under an electric field, thereby minimizing membrane fouling (Doyen et al. 2011). The system utilizes cation and anion exchange membranes in conjunction with UF membranes. Peptide migration from snow crab hydrolysates increased with peptide concentrations up to 4%. Doyen et al. (2012) selected 50 kDa membranes under a 14 V/cm electric field, which performed better than 20 kDa membranes at 2 V/cm. A positive correlation was observed between peptide migration and membrane MWCO (5–300 kDa), though selectivity for low‐MW peptides declined. Firdaous et al. (2009) showed that ultrafiltration at pH 3 using a 10 kDa membrane enhanced the concentration of antihypertensive VW peptides from alfalfa. Doyen et al. (2012) used 20–50 kDa UF membranes to selectively retain cationic and anionic peptides from flaxseed and salmon hydrolysates. Durand et al. (2020) applied a four‐UF membrane EDUF system for fractionating herring milt hydrolysates into antioxidant and anti‐inflammatory peptides. Suwal (2015) identified bioactive peptides such as ALPMHIR in whey hydrolysates obtained via EDUF. Przybylski et al. (2020) reported that peptide selectivity and purity up to 56.1% depended significantly on pH, with pH 9 favoring the isolation of antimicrobial peptide α137–141 using a 10 kDa membrane.

#### 5.3.6 Comparative Evaluation of Membrane‐Based Strategies for Peptide Recovery

Membrane‐based technologies, including UF, EMR, NF, and EDUF, offer distinct mechanisms for the efficient recovery of bioactive peptides:

- *UF* provides size‐based separation with relatively simple operation.
- *EMR* enhances hydrolysis yields via simultaneous enzymatic reaction and separation.
- *NF* excels in charge‐based fractionation using electrostatic interactions.
- *EDUF* offers high specificity and minimal fouling through electric field‐assisted transport.

Collectively, these strategies illustrate that peptide production at scale can be significantly optimized by selecting appropriate membrane types, feedstock characteristics, and operational parameters. Future advancements in membrane design and process integration will further drive the scalability and functional efficiency of peptide bioseparation systems (Alavi and Ciftci 2023).

## 6 Properties of Bioactive Molecules From Fish By‐Products

### 6.1 Solubility and Bioavailability

Solubility is a primary factor influencing the absorption and bioavailability of peptides and proteins. Peptides with low molecular weights (typically < 1 kDa) are generally more soluble and exhibit enhanced gastrointestinal absorption compared to higher molecular weight peptides (Kurnianto et al. 2024). This property is particularly important for its application in nutraceuticals and functional foods.

### 6.2 Stability and Processing Methods

The stability of bioactive peptides is significantly influenced by environmental factors such as pH and temperature. Enzymatic hydrolysis, a commonly used method for peptide production, tends to preserve the structural integrity and functionality of the resulting peptides. In contrast, chemical hydrolysis often leads to denaturation and degradation, resulting in reduced bioactivity (Ishak and Sarbon 2018). Hence, the method of hydrolysis plays a key role in maintaining the functional properties of peptides.

### 6.3 Molecular Size and Antioxidant Activity

Molecular size is another crucial determinant of bioactivity. Peptides with molecular weights between 500 and 1000 Da have demonstrated superior antioxidant activities due to their enhanced ability to donate protons and scavenge reactive oxygen species efficiently (Ishak and Sarbon 2018). These smaller peptides can also penetrate biological membranes more readily, increasing their functional potential.

### 6.4 Charge and Biological Interactions

The net charge and distribution of amino acids in a peptide influence its interaction with cellular membranes and pathogens. Positively charged peptides, particularly those derived from fish protein hydrolysates, exhibit strong antibacterial activity, especially against Gram‐positive bacteria, by disrupting bacterial membranes (Ennaas et al. 2015). This makes them promising candidates for antimicrobial applications.

## 7 Bioactive Properties and Applications

Bioactive peptides of fish byproducts show a wide range of antioxidant and bioactive properties including antihypertensive, antihyperglycemic, anticancer, and antimicrobial, Table 4 illustrates the bioactive properties of fish bioactive peptides.

**TABLE 4:** Pharmaceutical properties and applications of fish‐derived bioactive compounds.

Fish species	Raw material	Enzyme/hydrolysis method	Key pharmaceutical properties	Applications	References
Tuna (*Thunnus albacore*)	Byproducts	Not specified	Diminishes ACE and aldose reductase activity (IC_50_: 41.5–70.4 μg/mL)	Blood pressure and diabetes control	Wijesekara et al. (2011)
Crucian carp ( *Carassius carassius* )	Muscle	Alkaline protease (122 U/mL, 39°C, pH 11, 10 h)	Inhibits porcine pancreas lipase (53.04%) and α‐amylase (20.03%)	Antidiabetic food additive	Liu et al. (2013)
Sturgeons ( *Acipenser schrencki* )	Skin	Pepsin (37°C, pH 2.0), trypsin (37°C, pH 7.2), alcalase (50°C, pH 8.0)	Peptides reduce ACE activity (IC_50_: 3.47 μg/mL)	Antihypertensive supplement	Zou et al. (2014)
Half‐fin anchovy ( *Setipinna taty* )	Mince	Pepsin (1100 U/g, pH 2.0, 2.4 h)	Synthetic peptide YALRAH inhibits prostate cancer cells (PC‐3) with IC_50_ 8.1 mg/mL	Anticancer agent for prostate cancer	Song et al. (2014)
Tuna fish ( *Thunnus tonggol* )	Cooking juice	Protease XXIII (2.1%, 6 h)	Inhibits breast cancer cells (MCF‐7) with IC_50_ 1.39 mg/mL for > 2.5 kDa hydrolysate	Anticancer agent for breast cancer	Hung et al. (2014)
Bluefin leatherjacket (*Navodon septentrionalis*)	Head	Papain (3:20 w/w, 50°C, pH 7.0, 5 h)	Peptides (WEGPK, GPP, GVPLT) show DPPH (EC_50_: 1.927–4.541 mg/mL), ABTS (EC_50_: 2.472–5.407 mg/mL) scavenging	Antioxidant food ingredient	Chi, Wang, Wang, et al. (2015)
Croceine croaker ( *Pseudosciaena crocea* )	Mince	Papain (1%, 50°C, pH 7.0, 4 h), alcalase (2%, 50°C, pH 9.5, 4 h)	Radical scavenging (IC_50_: 1.35–5.01 mg/mL)	Antioxidant agent	Chi, Hu, et al. (2015)
Giant grouper ( *Epinephelus lanceolatus* )	Roe	Protease N (1:100 w/w, 50°C, pH 8, 9 h)	Inhibits oral cancer cell growth (Ca9‐22: IC_50_ 0.85 mg/mL; CAL 27: undetectable)	Potential anticancer agent for oral cancer treatment	Yang et al. (2016)
Tilapia (*Oreochromis* sp.)	Scale	Not specified	Improves insulin response and glucose tolerance	Antidiabetic agent	Iba et al. (2016)
Smooth hound ( *Mustelus mustelus* )	Visceral mass (stomach, intestine)	Neutrase (6/1 U/mg, pH 7.0), purafect (6/1 U/mg, pH 10.0)	Peptides (e.g., GPAGPRGPAG) inhibit ACE (IC_50_: 74.99–90.54 μg/mL)	Blood pressure management	Abdelhedi et al. (2016)
Salmon ( *Salmo salar* )	Trimmings	Corolase PP	Strongly inhibits DPP‐IV (IC_50_: 0.08 mg/mL)	Antidiabetic supplement	Neves et al. (2017)
Seabass ( *Lates calcarifer* )	Skins	Alcalase (1–2 units/g, 55°C, 3 h)	Reduces colon (Caco‐2) and liver (HepG2) cancer cell growth to 39% at 25 mg/mL	Anticancer agent for colon and liver cancer	Sae‐Leaw et al. (2017)
Chinese giant salamander ( *Andrias davidianus* )	Muscle	Alcalase, flavourzyme, trypsin	Inhibits α‐amylase and α‐glucosidase (IC_50_: 2.86 × 10^3^ μg/mL and 42.93 μg/mL for Leu‐Gly‐Gly‐Gly‐Asn)	Antidiabetic health product	Ramadhan et al. (2017)
Anchovy (not specified)	Fish meal	Alkaline protease (0.8 L, 55°C, pH 8.0, 3 h)	Exhibits ferrous ion chelating (36.87%) and superoxide scavenging (26.91%–37.85%)	Antioxidant supplement	Wang et al. (2018)
Tuna (not specified)	Byproduct	Prolyve BS (1.25%, 50°C, pH 7, 1 h)	DPPH scavenging (IC_50_: 0.32–0.66 mg/mL), superoxide scavenging (IC_50_: 0.115–0.53 mg/mL), and reduces lipid oxidation	Antioxidants in food preservation	Saidi et al. (2018)
Blue whiting ( *Micromesistius poutassou* )	Mince	Alcalase 2.4 L, flavourzyme 500 L (0.74% v/w, 50°C, pH 7, 4 h)	Increases insulin and GLP‐1 secretion, reduces DPP‐IV activity	Antidiabetic ingredient	Harnedy et al. (2018)
Asian swamp eel (*Monopterus* sp.)	Flesh	Alcalase (2.26%, 50.18°C, pH 7.89, 84.02 min)	Inhibits breast cancer cells (MCF‐7) with IC_50_ 6.50 μg/mL for 3 kDa hydrolysate	Anticancer agent for breast cancer	Halim et al. (2018)
Boarfish ( *Capros aper* )	Muscle	Alcalase 2.4 L, flavourzyme 500 L (0.67%, 50°C, pH 7.0, 4 h)	Inhibits DPP‐IV (IC_50_: 1.18 mg/mL), boosts insulin and GLP‐1 secretion, enhances glucose tolerance	Antidiabetic functional ingredient	Parthsarathy et al. (2019)
European seabass ( *Dicentrarchus labrax* ), Gilthead seabream ( *Sparus aurata* )	Not specified	Alcalase (0.5%–2%, 60°C, pH 7.5, 2 h), α‐chymotrypsin (0.5%–2%, 45°C, pH 8, 2 h)	Promotes kidney cell proliferation (MDCK1: 147%) and reduces colon cancer cells (HT‐29: 40%–60%)	Anticancer and cell proliferation agent	Altınelataman et al. (2019)
Kawakawa ( *Euthynnus affinis* )	Muscle	Skipjack tuna pepsin (2%, 37°C, pH 2.0)	Provides antioxidant effects	General antioxidant use	Taheri and Bakhshizadeh (2020)
Kawakawa ( *Euthynnus affinis* )	Muscle	Skipjack tuna pepsin (2%, 37°C, pH 2.0)	Inhibits ACE (IC_50_: 0.44–1.94 mg/mL across fractions FPH I–IV)	Antihypertensive agent	Taheri and Bakhshizadeh (2020)
Mackerel ( *Scomber japonicus* )	Muscle	Protamex (2%, 50°C, 110 rpm, 1 h)	Peptides show DPPH scavenging (36.34%) and SOD‐like activity (28.94%)	Antioxidant food additive	Bashir et al. (2020)
Raw sardines (*Sardine pilchardus*)	Muscle	Subtilisin, trypsin, flavourzyme mixture	Inhibits DPP‐IV (IC_50_: 1.83 mg/mL)	Antidiabetic therapeutic agent	Hong et al. (2020)
Round scad ( *Decapterus maruadsi* )	Whole dried powder	Alcalase (8000 U/g, 55°C, pH 9.5)	DPPH scavenging (30.25%), reducing power (0.276), ORAC (75.14 μmol/L)	Antioxidants in functional foods	Chen et al. (2020)
Baltic herring ( *Clupea harengus* )	Muscle and byproducts	Alcalase (2%, 55°C, 3 h), flavourzyme (1%, 55°C, 1 h)	Inhibits lung (A549) and colon (HCT8) cancer cells (IC_50_: 149.5–839.5 μg/mL)	Anticancer agent for lung and colon cancer	Mäkinen et al. (2022)
Sturgeon ( *Acipenser sinensis* )	Muscle	Papain (3.5%, 70°C, pH 6, 6 h), alcalase (3.5%, 55°C, pH 8.5, 6 h)	DPPH (IC_50_: 3.15–3.64 mg/mL) and ABTS (IC_50_: 1.58–1.92 mg/mL) scavenging	Antioxidant health supplement	Noman et al. (2022)
Tuna (*Thunnus albacore*)	Trimmings	Alcalase (3000 U/g, 50°C, pH 8.0, 4 h)	Reduces tumor volume in male BALB/c mice	Anticancer agent in animal models	Zhao et al. (2023)

### 7.1 Bioactive Properties

#### 7.1.1 Antioxidant Properties

Oxygen plays a significant role in facilitating cellular respiration and energy metabolism in organisms. As a result of oxidation, the increased level of aerobic metabolism is rather disadvantageous since it produces reactive oxygen species (ROS) and free radicals as waste products. These reactive molecules can cause much damage in the cell by oxidizing proteins and unsaturated fatty acid chains, thus causing tissue injury. Oxidative stress is characterized by the generation of ROS in an amount that is above the normal capacity of the body's antioxidant system or any mechanism that hinders it. There is a decrease in the ratio that leads to lipid peroxidation, degradation of proteins, fragmentation of DNA, and inactivation of the enzyme. Likewise, free radicals can cause harm to biological molecules like cellular lipids, proteins, and DNA. The ROS molecules are uncontrolled and known to provoke several diseases, ranging from diabetes, neurodegeneration, cancer, to inflammation, etc. Recently, FPH peptides obtained from separated fish by‐products have received interest for their antioxidant activity. These bioactivities are mainly associated with peptides according to the protein structure of FPH. The release of such peptides occurs because of endogenous or exogenous enzymatic hydrolysis, and they are usually small in size (comprising 5–16 amino acids) and possess amino acids such as proline (Pro) or tyrosine (Tyr). For instance, Tang et al. (2018) described the ability of peptides with a molecular weight less than 500 Da derived from Round Scad ( *Decapterus maruadsi* ) to inhibit the xanthine oxidase enzyme. Li, Feng, et al. (2022) obtained peptide VQVLAGPVVKLY from grass carp scale hydrolysate that lowered the degree of lipid peroxidation in a linoleic acid system to a great extent. These studies provide evidence that amino acid sequence and molecular weight are important factors that affect the functionality of antioxidants. Nikoo, Regenstein, Haghi Vayghan, and Walayat (2023) noted that enzymatic hydrolysis helps to increase the amount of antioxidant peptides and exposure of the peptides to the free radicals and metal ions. Furthermore, peptides assumed other functions, for instance, inhibitory effects on ice crystal formation and overcoming protein oxidation, which enhance the cryopreservative properties (Nikoo et al. 2016). Nikoo et al. (2016) determined and compared four enzymes in the process of producing antioxidant silver carp fin protein hydrolysates (SCPH). Protein hydrolysates obtained by trypsin and alcalase had positive effects in both the ABTS radical cation scavenging and ferrous ion chelation. In the same studies, Naghdi et al. (2023) extracted antioxidant peptide fractions of < 3 kDa using alcalase to use them in combination with alginate‐starch coatings able to extend the shelf life of refrigerated rainbow trout fillets. In their study, Hamzah et al. (2021) investigated the effectiveness of SCPH in decreasing lipid oxidation in fish emulsion sausage regarding peroxide values and TBARS. Cod skin peptides reduce cellular oxidation by 40% (Wang et al. 2016), and salmon head hydrolysates exhibit an IC_50_ of 0.87 mg/mL for ACE inhibition (Zhang et al. 2021), supporting their use in functional foods, though clinical trials are needed (Stevens et al. 2018). Similarly, Parvathy et al. (2018) observed that the use of tuna waste protein hydrolysate at 0.5% concentration as applied in the preservation of dressed sardines in ice slowed down the rate of oxidation.

Recently, Nurilmala et al. (2020) have also isolated potent free radical scavenging peptides with a molecular weight of less than 3 kDa from yellowfin tuna skin, and the results support the antioxidant activity of small peptides. Li et al. (2019) confirmed that the tilapia skin collagen polypeptides with a molecular weight below 3 kDa have a potential therapeutic effect on oxidative stress‐induced liver and kidney injury in mice.

Dey and Dora (2014) showed that as much as 5 mg/mL of seafood waste protein hydrolysates could enhance croaker fish fillets by reducing thiobarbituric acid (TBA) levels during storage at 4°C. Je et al. (2007) isolated and characterized an inactive peptide named VKAGFAWTANQQLS from tuna backbone protein and found that it scavenged DPPH, hydroxyl, and superoxide radicals in a dose‐dependent manner.

Chalamaiah et al. (2012) and Kou et al. (2013) pointed out that antioxidant peptides operate on the principle of reduction of hydroperoxides, free radicals, ROS, and sequestration of metals. When it comes to structure and consequently bioactivities, the influencing factors include peptide size, amino acid sequence, and terminal residues (Elias et al. 2008). Some peptides also influence endogenous antioxidant enzymes such as superoxide dismutase and glutathione peroxidase activity (Mehta and Gowder 2015). Ben Khaled et al. (2012) revealed that antioxidant enzyme activity and HDL cholesterol levels were elevated while malondialdehyde (MDA) decreased in sardinelle protein hydrolysate‐fed rats. Tilapia hydrolysates were shown to have reduced lipid hydroperoxides and TBA‐reactive species (TBARS) in stored mahi‐mahi red muscle.

**FIGURE 3:**
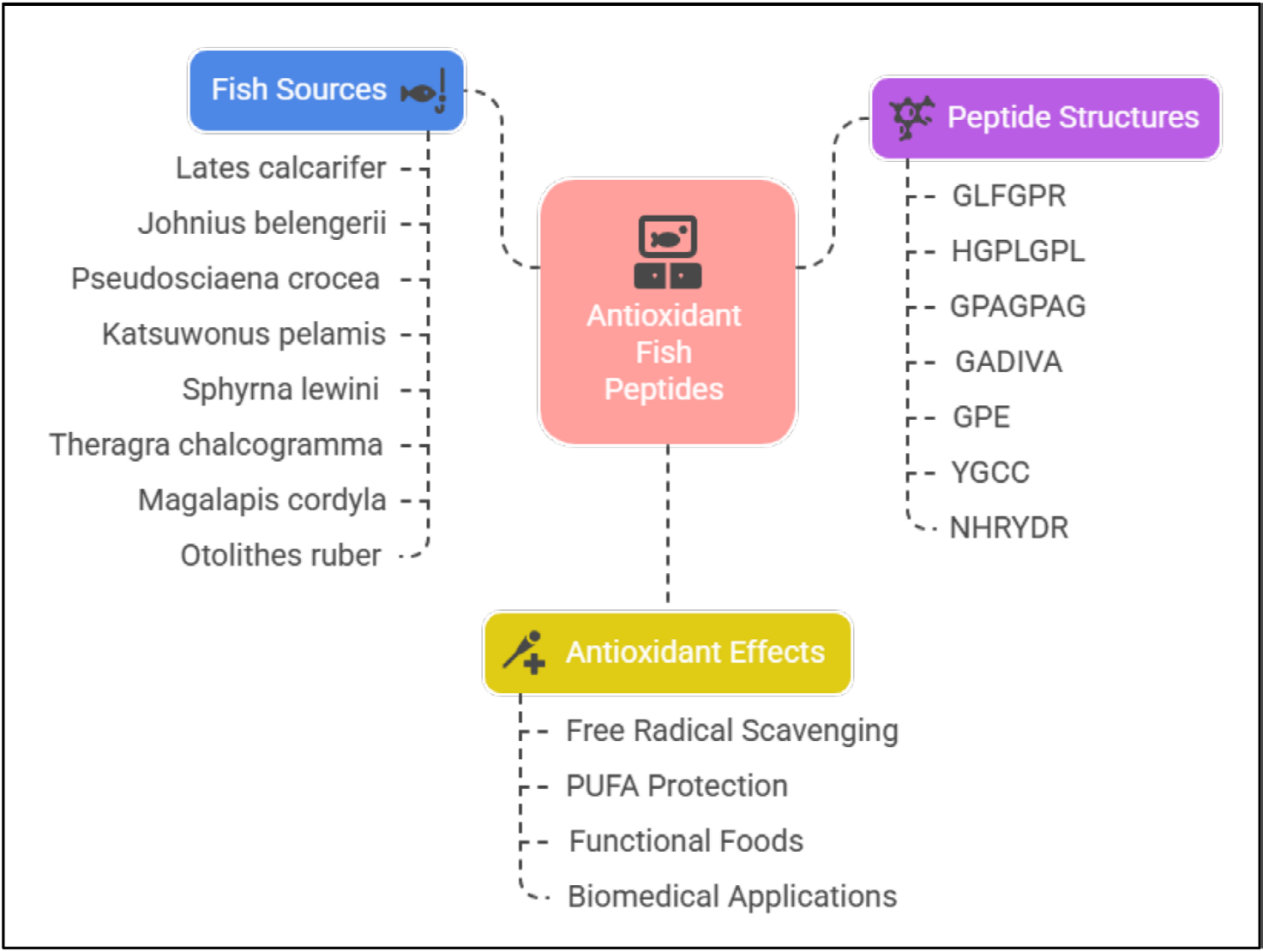
Fish species, their derived antioxidant peptides, and their biological effects.

Different types of antioxidant peptides have been isolated from several fish and fishery resources. For instance, Sae‐leaw et al. (2016) extracted GLFGPR from *Lates calcarifer* skin; Mendis et al. (2005) obtained HGPLGPL from *Johnius belengerii* skin; and Wang et al. (2013) identified GPAGPAG, GFPSG, and GLFGPR from the scales of *Pseudosciaena crocea* fish (Table 2). Qiu et al. (2019) reported the isolation of antioxidant peptides GADIVA and GAEGFIF from *Katsuwonus pelamis* bones. Subsequently, peptides ranging from GPE, GARGPQ, GFTGPPGNG have been isolated from *Sphyrna lewini* cartilage and skin (Li et al. 2017) as well as YGCC, DSSCSG, NNAEYYK, and PAGNVR from *Theragra chalcogramma* skin (Sun et al. 2016). Sampath Kumar et al. (2012) reported the identification of peptides NHRYDR and GNRGFACRHA from *Magalapis cordyla* and *Otolithes ruber* that resisted the peroxidation of polyunsaturated fatty acids. Altogether, these papers generated evidence on the antioxidant effects of fish protein hydrolysates obtained from various sources and using different processing methods. The ability to combat free radicals depends on some aspects, such as peptide structure, enzymatic origin, and molecular size, proving the effectiveness of peptides in functional foods and biomedical sectors. Referring to the current literature, many in vitro and animal investigations are available, although human clinical trials are still lacking and are crucial before determining their applicability in health management and food preservation. Figure 3 illustrates fish species, their derived antioxidant peptides (with specific sequences), and the biological effects of these peptides.

#### 7.1.2 Antihypertensive Activity

Cardiovascular diseases are responsible for over 30% of global mortality worldwide due to severe hypertension, which has been identified as one of their causal factors (Lee and Hur 2017). Within the renin‐angiotensin system that controls mammalian blood pressure, renin converts angiotensinogen to angiotensin I, which is acted upon by angiotensin I converting enzyme (ACE) to form the physiologically active angiotensin II (Aluko 2019). Angiotensin II is a strong vasoconstrictor, and excessive levels lead to hypertension by neutralizing the effects of potent vasodilators that act to lower blood pressure. ACE can also hydrolyze and neutralize bradykinin, a potent vasodilator. Consequently, hypertension can be controlled by the reduction of ACE activity and inhibition of bradykinin breakdown in blood vessels. However, synthetic ACE inhibitors, such as drugs, produce unwanted side effects despite being effective in managing conditions. Therefore, while seeking other ways to manage hypertension, food‐derived ACE inhibitory peptides have been considered as a viable option to these drugs (Li, Feng, et al. 2022). With respect to these peptides, YLRLHF was found to be one of the most potent, with an IC_50_ value of 121.90 μmol/L for ACE inhibition. The molecular docking also showed multiple contacts such as hydrogen bonds, Zn^2+^ binding, and π‐π stacking with His421, which indicate binding of the peptide to the active site of ACE to block catalysis (Masuyer et al. 2012).

Thus, protein hydrolysates from fish by‐products are potential sources of ACE‐inhibitory peptides. For example, Auwal et al. (2019) applied pineapple protease to break down fish protein obtained from Stonefish ( *Actinopyga lecanora* ). Five peptides of molecular weight less than 1000 Da were analyzed and identified to be most effective, of which ALGPQWY (794.44 Da) had the strongest activity with an IC_50_ value of 0.012 mM. The outcomes obtained indicate that fish waste‐derived peptides may be used as antihypertensive therapeutic agents.

Another similar study carried out by Yu et al. (2020) showed that GHVGAAGS (600–1000 Da) isolated from fish collagen has vital ACE inhibitory activity. The interaction analysis of macromolecules showed that GLN281, HIS353, LYS511, and HIS513 are responsible for the interaction that leads to the ACE inhibitory effect, exhibiting an IC_50_ of 407.3 μM. Subsequently, Barkia et al. (2019) research also identified the ACE inhibitory activities of more indigenous marine diatoms, namely, Bellerochae and Nitzschia, with activities of 84% and 61%, respectively. This activity was related to the generation of small molecular peptides by enzymatic degradation. In the same way, Barkia et al. (2019) suggested that peptides in fermented sausages produced by mixed microbial culture using *Lactobacillus plantarum* and *Staphylococcus simulans* had improved ACE inhibitory effect, which showed that microbial hydrolysis in food matrices has synergistic effects. Khositanon et al. (2021) studied the ACE inhibitory peptides from enzymatically hydrolyzed fish sauce industrial waste and emphasized the potential of marine by‐products for the synthesis of bioactive peptides. The underlying mechanism of action of these peptides is also in the suppression of the formation of an active angiotensin II and inhibition of bradykinin degradation (Admassu et al. 2018). Several studies reaffirm that the ACE inhibitory potential is inversely proportional to the molecular mass of peptides, and smaller peptides with less than 1000 Da proved to be most potent. The in vitro findings of ACE‐inhibitory activities have also been validated using animal model studies to support antihypertensive potential. For example, Lin, Chen, Jin, et al. (2019) and Lin, Chen, Tsai, and Chen (2019) identified four peptides from the skin of cobia fish ( *Rachycentron canadum* ) in which oral administration was found to significantly lower the systolic and diastolic blood pressure of spontaneously hypertensive rats (SHRs) by 21.9 and 15.5 mmHg, respectively and this effect lasted up to 4 h, While there are numerous experiments to show the potential antihypertensive effects of fish peptides, there is a poor application of these results to human use because most of the tests have been carried out using vitro assays and animal feeding experiments. Therefore, further clinical research studies must be conducted to determine the efficacy and the beneficial and undesirable effects of these peptides on human beings.

#### 7.1.3 Anticancer Properties

With increasing life expectancy, changing dietary habits, and growing environmental pollution, cancer has become one of the leading causes of death worldwide, surpassing even cardiovascular diseases (Feigin 2017). Cancer development is closely linked to morphological and genetic alterations at the DNA level, often triggered by permanent gene mutations. According to the *Progress Against Cancer, 2022* report released by the American Association for Cancer Research (AACR), global cancer incidence is projected to rise to approximately 28 million by 2040, with an expected mortality of 16.2 million (Williams et al. 2022). Between August 2022 and July 2023, the U.S. FDA approved 14 new anticancer therapeutics, two new imaging agents, and expanded indications for 12 previously approved drugs. Despite these advancements, conventional anticancer therapies often cause severe side effects, prompting the search for safer, cost‐effective alternatives. In this context, natural compounds, particularly bioactive peptides and protein hydrolysates derived from marine organisms, have emerged as promising candidates due to their selective cytotoxicity and minimal side effects. Numerous in vitro studies have demonstrated the anticancer potential of peptides and hydrolysates from marine sources. For instance, Chen et al. (2023) reported that fishbone fermented with *Monascus purpureus* (FBF) significantly inhibited the proliferation of HCT‐116 colorectal cancer cells. This inhibition was achieved through S‐phase and G2/M‐phase arrest, induction of apoptosis, and autophagy, highlighting FBF as a potential therapeutic agent for colorectal cancer. Similarly, Kandyliari et al. (2020) found that FPHs derived from *Argirosomus regius* and *Sparus aurata* by‐products exhibited inhibitory effects on COLO320 human colorectal adenocarcinoma cells at concentrations ranging from 0.5 to 1 g/L.

#### 7.1.4 Confirmed In Vitro Efficacy of Marine Peptides

Several individual marine peptides have shown marked anticancer effects in cell line studies. For example, Deepak et al. (2020) identified the cyclic dipeptide *cyclo*(*L‐leucyl‐L‐prolyl*) from marine sources, which reduced the viability of triple‐negative breast cancer (TNBC) cell lines MDA‐MB‐231 and MDA‐MB‐468 by inducing DNA damage and apoptosis. Likewise, Bosseboeuf et al. (2019) discovered that the pyroglutamate‐modified peptide pE‐K092D, isolated from the testis of the less‐spotted dogfish, significantly inhibited the growth of MDA‐Pca‐2b prostate cancer cells by affecting cell proliferation and inducing necrosis.

#### 7.1.5 Antimicrobial Activity

The abuse of antibiotics in human beings, livestock, and fish farming has contributed to the emergence of antibiotic‐resistant bacteria. Consequently, there has been a substantial interest in the development of novel drugs, namely antimicrobial peptides (AMPs), which combine a broad‐spectrum acting profile with multiple methods of action, as pointed out by Felício et al. (2017). As opposed to conventional antibiotics that directly affect one bacterial component, many AMPs simultaneously target various components in a pathogen, and thus, microbial resistance cannot be quickly developed. It has been shown that the functional properties of peptides, especially those from protein hydrolysates, depend on the conditions used during enzymatic hydrolysis. For example, it was reported that the Protamex hydrolysate of tuna head proteins exhibited higher bactericidal activity against *Listeria monocytogenes* and *Escherichia coli*, while the Pancreatin hydrolysate showed higher bactericidal activity against *Staphylococcus aureus* and Enterococcus, implying the specific enzyme‐based generation of bioactive peptides (Abd El‐Rady et al. 2023).

Ulzanah et al. (2023) showed that collagen peptides from the skin of tilapia, milkfish, and catfish effectively inhibited *Aeromonas hydrophila* and may be employed as a biocontrol approach in aquaculture. The antimicrobial effect of these peptides has been suggested to be related to the hydrophobic and cationic characters that bring about disturbance of microbial membranes. Fermented fish by‐products are also a prospective source of AMPs. Consequently, Malaysian fish intestinal by‐products fermented using *Lactobacillus casei* strains LC216 and LC217 considerably prevented the growth of *Listeria monocytogenes, Salmonella typhimurium, Escherichia coli*, and *Listeria innocua*. This was because there are instances where the hydrophobic amino acid residues would form a barrier, thus supporting the argument that biochemical composition determines the functionality of AMP.

From a mechanism of action, fish protein‐derived AMPs have been reported to cause disruption of bacterial membranes, inhibition of nucleic acid synthesis, and alteration of immune defense, thus providing bacteriostatic and antiviral properties (Barbosa et al. 2020). Chauhan et al. (2020) divided these AMPs based on structural characteristics: disulfide‐bridged cyclic membrane‐active peptides, Gly‐Pro/His antibody‐rich membrane‐active peptides, and linear helical membrane‐active peptides, as they affect the interaction with microbial membranes and their intracellular targets. Later, the antibacterial properties of the peptides from *Psenopsis cyanea* showed significant inhibition against both Gram‐positive and Gram‐negative bacteria, particularly *Aeromonas hydrophila* . More about AMPs was explained by others, namely, the mechanisms regarding the formation of pores in biochemical membranes and intracellular targeting that does not involve the lysis of the cell (Lee et al. 2024). Semreen et al. (2018) further stated that the AMPs synthesized in bacteria and eukaryotes are active against viruses, fungi, protozoa, and parasites.

In comparison to antibiotics, which usually have a single mode of action that enhances microbial resistance, AMPs act through multiple pathways and thus are better suited for combating antimicrobial resistance (Felício et al. 2017). Due to their origin, structure, and functions, current and prospective AMPs, especially marine and protein hydrolysate libraries, can be applied in a variety of therapeutic as well as food industries.

#### 7.1.6 Antihyperglycemic Activity

The global increase in diabetes is predicted to reach about 700 million adults by 2045; therefore, there is a need to have more effective and safer therapeutic agents (Rivero‐Pino et al. 2020). Previous studies have mainly focused on the impact that fish‐derived peptides have on numerous diabetic measurements. Bhatnagar and Mishra (2022) focused on α‐glucosidase as a therapeutic agent because this enzyme aids carbohydrate digestion in humans. Diabetics can manage their post‐meal blood glucose levels more easily by using agents that block this enzyme, which represents a key aspect of diabetes management. Munawaroh et al. (2024) examined fish peptides in salmon gelatin (VW) and tilapia gelatin (WF), which showed strong α‐glucosidase inhibitory action for potential use as antihyperglycemic agents.

The DPP‐IV inhibitory activity of Alcalase and Flavourzyme‐treated Baltic Herring ( *Clupea harengus membras* ) hydrolysates reached IC_50_ values of 5.38–7.92 mg/mL, which demonstrates potential clinical applications (Mäkinen et al. 2022). The systemically prepared hydrolysates through Selar ( *Selar crumenophthalmus* ) demonstrated the potential to increase GLP‐1 secretion in rats, according to the results obtained by Kusuma et al. (2021), through Tempeh protease application. Natsir et al. (2019) examined how peptide substances derived from yellowfin tuna ( *Thunnus albacares* ) bone collagen blocked α‐glucosidase activity, thus making the peptides suitable for postprandial hyperglycemia management. These research reports demonstrate how the inhibition of DPP‐IV and α‐glucosidase enzymes serves as a therapeutic approach to controlling type 2 diabetes. The technological evidence demonstrates that fish protein hydrolysates exhibit an incretin effect through GLP‐1 GIP and enteroglucagon enhancement together with DPP‐IV inhibition (Sharkey et al. 2020). Various mechanisms supported the argument that natural products could potentially serve as alternative hypoglycemic medications. Previous research indicated that protein hydrolysates of *sardine pilchardus* showed maximum DPP‐IV inhibitory activity for the peptides below 1400 Da, especially YACSVR and NAPNPR sequences (Rivero‐Pino et al. 2020). According to Gong et al. (2020), *Stichopus japonicas* peptides showed their ability to increase glucose uptake in mature 3T3‐L1 adipocytes through DPP‐IV inhibition. Atlantic salmon ( *Salmo salar* ) skin and trimmings‐derived peptides were also shown to activate GLP‐1 release for improved insulin production (Harnedy et al. 2018a).

These peptides and protein hydrolysates modulate the essential enzymes and hormones involved in type 2 diabetes management, including α‐glucosidase, DPP‐IV, and GLP‐1 (Yu et al. 2025). The current available in vitro and in vivo data look promising, yet clinical data remain insufficient. Therefore, human clinical trials are needed to determine the number of fish peptides available for human use, along with their recommended dose levels and health effects, before the industry can fully adopt them in biomedical products and functional foods.

#### 7.1.7 Structure‐Bioactivity Relationship of Peptides

Research has confirmed that structure directly affects various bioactive properties of peptides. A protein known as a peptide contains amino acids that possess four components, namely hydrogen bond donors (>N—H), acceptors (>C=O), an amino‐terminal (coupled with the amino group), and a carboxyl‐terminal (—COOH group). The anti‐microbial properties of cyclic peptides: oligopeptide, lipopeptide, glycopeptide, depsipeptide polymers have been observed to be related to the helix, sheet, and turn secondary structures (Koehbach and Craik 2019). Oh et al. (2020) demonstrated that *Mytilus coruscus* peptides benefit from helical structure along with hydrophobic and hydrophilic regions combined with Arg/Leu modifications, which enhance antimicrobial activity. Wang et al. (2016) showed that pore‐forming ability represents the main determinant for antimicrobial activities in *Litoria genimaculata* peptides. Bioactive peptides (2–20 kDa) with high glycine and proline content exhibit enhanced antioxidant and antihypertensive properties (Karami and Akbari‐Adergani 2019).

TCA cycle antioxidant activity receives direct influence from both aromatic amino acids (Tyr, Trp, and Phe) together with histidine and hydrophobic amino acids such as Val, Leu, Ile, Pro, Ala, Met, and Gly (Harnedy et al. 2018b). The inhibitory interaction between ACE and IWW of cobia skin depends on hydrophobic, electrostatic, and hydrogen bond interactions (Lin et al. 2020). Both Yu et al. (2020) and Kong et al. (2020) revealed that the ACE inhibition capabilities of fish collagen peptides stem from hydrophobic residues like leucine and alanine interacting with LYS 511 and HIS 513 alongside GLN 281 and HIS 353. Anticancer effects occur most effectively for NAPNPR and YACSVR peptides from *Sardine pilchardus* (Rivero‐Pino et al. 2020). Anticancer activity occurs through cationic and moderately hydrophobic peptides together with the secondary structure for peptides that contain six residues in length (Chiangjong et al. 2020; Li and Wang 2016). The amino acid composition of salmon skin gelatin contains Glu, Ala, Ser, Gln, Gly, Asp, Leu, Met, and cationic Arg that amount to 12.7%, which favor GLP‐1 release and insulin secretion (Harnedy et al. 2018b). Additional research works are required to establish trends that describe bioactive properties of diverse fish protein‐derived peptides, because amino acid type and arrangement determine peptide activity.

### 7.2 Applications of Bioactive Molecules From Fish By‐Products

The utilization of fish waste includes fish oil (high in EPA and DHA, 10%–15% of fat) and chitosan for medical applications, especially for wound healing, in addition to being a source of essential minerals (Ghaly et al. 2013). Improved enzymatic hydrolysis and demineralization make it applicable for top‐shelf products (Lingam et al. 2023). As the fish filleting process disposes of 45% of the fish as waste materials, the application of this resource in biofuels, medicine, and functional food may bring sustainability and economic benefits; indeed, fish waste is now considered critical to biotechnology development (Caruso et al. 2020; Jasrotia et al. 2024). Table 6 illustrates the application of bioactive peptides in different industries.

Table 2 presents an analysis of the high‐value nutrients in fish by‐products and the value of the nutrients in the market, together with their uses. For instance, collagen from fish skins and bones has a market value of $ 9–14/kg and is applied in cosmetics and biomedical scaffolds (Silva et al. 2014). Hydroxyapatite from fish bones is important in bone tissue regeneration, while enzymes from fish viscera are used in the formulation of detergents and biocatalysis. These examples show the importance of fish by‐products in the economic and functional aspects of human life in the provision of bioactive molecules.

#### 7.2.1 Food Industry

Fish parts and by‐product components include proteins and peptides with lipids, which find application as functional food ingredients as well as food preservatives in the food supply chain (Table 5). The hydrolysates derived from enzymatic processes are incorporated into fortified crackers and protein bars (Siddik et al. 2021). Research indicates tuna cooking juice hydrolysates contain peptides with strong antioxidant capabilities that enable better product preservation during storage. The application of antimicrobial peptides together with chitosan films serves two main functions in food preservation methods. Studies show that chitosan acquired from crustacean shells and fish scales functions as a bioactive substance that inhibits the growth of different harmful organisms, including *Escherichia coli* and *Staphylococcus aureus* . Prepared edible films with chitosan help keep perishable foods fresher longer because the food‐grade biopolymer reduces microbial action, together with lipid deterioration. Fish skin and bone residues yield gelatin, which functions as a texturing agent for gels and emulsion‐making processes and confectionery products because of its gelation and stabilization features (Silva et al. 2014).

**TABLE 5:** High‐value components in fish by‐products.

Component	Source	Market value (USD/kg)	Applications	Extraction method	References
Collagen	Skins, scales, bones	$9–14	Cosmetics, wound healing, tissue engineering	Enzymatic hydrolysis, acid/alkaline solvents	Silva et al. (2014)
Gelatin	Skins, bones	$8–12	Food gels, emulsifiers, and pharmaceutical capsules	Thermal denaturation, enzymatic hydrolysis	Ferraro et al. (2013)
Hydroxyapatite	Bones, scales	Not available	Bone grafts, dental implants, biomedical scaffolds	Calcination, enzymatic methods	He et al. (2019)
Enzymes	Viscera, guts	$50–100 (cod proteases)	Probiotics, food processing aids, detergents	Fermentation, enzyme‐assisted extraction	Kurnianto et al. (2024)
Polyunsaturated fatty acids (PUFAs)	Liver, viscera	$24–30 (cod liver oil)	Nutraceuticals, dietary supplements, infant formula	Supercritical fluid extraction, pressing	Blondeau (2016)
Chitin/chitosan	Shells, exoskeletons	$20–30	Biodegradable packaging, antimicrobial agents, and wastewater treatment	Chemical deproteinization, demineralization	Ciriminna et al. (2017)
Bioactive peptides	Protein hydrolysates	$10–20	Antioxidants, antihypertensive agents, functional foods	Enzymatic hydrolysis, fermentation	Korkmaz and Tokur (2022)
Minerals (calcium, phosphorus)	Bones, fins	Not available	Food fortification, dietary supplements, and biomedical applications	Acid leaching, calcination	Harikrishna et al. (2017)
Hyaluronic acid	Eyes, skin	$500–1000	Anti‐aging creams, joint health supplements, and ophthalmic solutions	Enzymatic extraction, fermentation	Zhang et al. (2019)
Amino acids	Protein hydrolysates	$10–50	Nutritional supplements, flavor enhancers, animal feed	Enzymatic hydrolysis, microbial fermentation	Siddik et al. (2021)
Vitamins (A, D, E)	Liver, viscera	$50–100	Dietary supplements, fortified foods, and skincare products	Solvent extraction, supercritical CO_2_	Dubazana (2022)
Protein hydrolysates	Frames, heads	$10–15	Sports nutrition, infant formulas, and anti‐inflammatory agents	Enzymatic hydrolysis, ultrafiltration	Li and Zhu (2024)

#### 7.2.2 Nutraceutical Applications

The pharmaceutical and nutraceutical industries widely accept fish by‐products since they possess numerous bioactive molecules that serve medical purposes. The two medically recognized bioactive omega‐3 fatty acids that benefit heart health are DHA and EPA. The scientific community recognizes omega‐3 fatty acids as a valuable therapeutic option to lower triglycerides because this reduces cardiovascular diseases; therefore, scientists have recommended their inclusion in supplements. Omega‐3 fatty acids, notably EPA and DHA, have been used as nutraceuticals (e.g., cod liver oil) in Nordic countries and the UK for decades, supporting cardiovascular health (Rubio‐Rodríguez et al. 2010). Collagen peptides serve as another maritime source that originates from fish bones alongside fish skins. Researchers have adopted specified peptides for boosting joint function, along with accelerating human body recovery. The application of collagen peptides as skin elasticity and hydration agents provides dermatological options as described by Felician et al. (2018). The peptides extracted from bonito fish demonstrate ACE inhibitory properties, which have the potential for use in treating high blood pressure (Yuan et al. 2024).

#### 7.2.3 Cosmetic Industry

Green cosmetics obtain their bioactive molecules from fish by‐products. Anti‐age cream and gel contain protein collagen, along with antioxidant peptides, as two main elements that stimulate skin cell generation and erase wrinkles (Table 6). The peptides stimulate collagen synthesis and minimize free radical levels that could hurt the skin (Zhang et al. 2019). The beauty and skin industry primarily utilizes chitosan‐based products because this substance serves both as a moisturizer and an antimicrobial agent. Table 5 highlights collagen's $9–14/kg market value, driving its use in cosmeceuticals and wound healing (Silva et al. 2014). The protective barrier that forms on the skin through this substance makes the skin more hydrated and resistant to microbial infections and, therefore, suits sensitive skin needs (Ciriminna et al. 2017). The functionality of chitosan nanoparticles makes them ideal for releasing active ingredients in targeted areas, which results in improved outcomes of cosmetic formulations.

**TABLE 6:** Applications for bioactive molecules in different industries.

Industry	Bioactive molecule	Application	Mechanism/functionality	Examples	References
Food	Protein hydrolysates	Nutritional fortification, functional foods	Enhances amino acid profile, antioxidant activity	Fortified crackers, sports nutrition supplements	Kurnianto et al. (2024)
Food	Omega‐3 fatty acids	Dietary supplements, infant formula	Reduces inflammation, supports cardiovascular health	Cod liver oil, fortified dairy products	Blondeau (2016)
Nutraceuticals	Collagen peptides	Wound healing, joint health supplements	Promotes cell proliferation, improves skin elasticity	Topical creams, osteoarthritis treatments	Silva et al. (2014)
Nutraceuticals	Antihypertensive peptides	ACE‐inhibitory drugs for hypertension	Inhibits angiotensin‐converting enzyme	Bonito fish peptides, antihypertensive nutraceuticals	Korkmaz and Tokur (2022)
Nutraceuticals	Anticancer peptides	Cancer therapeutics	Induces apoptosis, inhibits tumor growth	Peptide‐based drugs, combination therapies	Ramakrishnan et al. (2024)
Cosmetics	Collagen	Anti‐aging creams, moisturizers	Stimulates collagen synthesis, reduces wrinkles	Anti‐wrinkle serums, facial masks	Zhang et al. (2019)
Cosmetics	Chitosan	Hair and skincare products	Moisturizing and antimicrobial properties	Shampoos, conditioners, acne treatments	Ciriminna et al. (2017)
Biomedical	Hydroxyapatite	Bone tissue engineering, dental implants	Mimics bone structure, promotes osteogenesis	Bone grafts, dental fillings	He et al. (2022)
Biomedical	Encapsulated peptides	Drug delivery systems	Controlled release enhances bioavailability	Liposome‐based therapeutics targeted cancer treatments	Zhang et al. (2019)
Agriculture	Protein hydrolysates	Biofertilizers	Provides essential nutrients, promotes plant growth	Crop fertilizers, soil enhancers	Ramakrishnan et al. (2024)
Environmental	Chitosan films	Biodegradable packaging	Reduces plastic waste, antimicrobial barrier	Edible films, food wraps	Martínez‐Alvarez et al. (2016)
Environmental	Enzymes	Wastewater treatment	Breaks down organic pollutants, improves water quality	Industrial effluent treatment, aquaculture wastewater management	Kurnianto et al. (2024)

#### 7.2.4 Biomedical Applications

The biomedical industry has discovered that tissue engineering, together with drug delivery systems from fish bioactive molecules, continues to be exceptionally vibrant. Fish skin and bone collagen scaffolds serve as damaged tissue replacement materials for skin, cartilage, and bone applications. The scaffolds create platforms with conditions that assist cell adhesion and support cell division as well as cell specialization activities (He et al. 2022). The extraction of hydroxyapatite from fish bones proves beneficial because researchers have confirmed its bone‐structure similarity to human bone tissue. Scientists have achieved successful outcomes through encapsulating peptides with liposomes or nanoparticles to enhance delivery to specific tissues. Chronic disease management might benefit from omega‐3 fatty acids that became incorporated into liposomes to boost stability and bioavailability (Zhang et al. 2019). Modern developments showcase how fish‐derived bioactive molecules demonstrate varied functionality during biomedical solution approaches to complex problems.

#### 7.2.5 Agricultural and Environmental Uses

Fish by‐product hydrolysate applications are becoming widespread across the agricultural sector because biofertilizers represent their most promising usage. Due to their nitrogen, fish peptides can function as organic fertilizers that strengthen plant nutritional needs as well as soil fertility (Tran et al. 2023). Research studies have proven that fish protein hydrolysates enhance agricultural crop yield while boosting their ability to handle environmental stress factors, including water scarcity alongside saline conditions.

The primary sectors that utilize bioactive molecules are described here. Protein hydrolysates serve as nutritional enhancers when producing chitosan films, which food companies use for food preservation applications.

Omega‐3 supplements and collagen peptides serve in pharmaceutical treatments by providing heart condition management while treating wounded tissue, respectively. The same pattern occurs for collagen and chitosan, which both appear in cosmetic products, particularly those designed to decrease aging signs and achieve moisture retention. The examples discussed highlight different product areas where bioactive compounds derived from fish are used.

Scientists verified the use of bioactive fish by‐product compounds within pharmaceuticals, along with food items, cosmetics, and biomedical products. Their varied capabilities and health‐friendly operations make them suitable for addressing major problems within food security, healthcare, and environmental protection. The successful resolution of current existing issues stands as a necessity for maximizing the use of these substances, particularly regarding extraction and standardization, along with scalability factors. Future research should focus on finding the optimal methods to merge green technological innovations with interdisciplinary cooperation for the absolute exploitation of these exceptional substances.

Table 4 shows some of the issues that must be addressed, including technical variation, cost, and regulatory risk, together with possible solutions. For instance, AI optimization and green extraction do well to deal with issues of scalability, while biorefinery and public‐private partnerships do well to deal with issues concerning the economy. Thus, ecological solutions in the form of waste‐free configurations and life cycle assessments (LCAs) are aimed at environmental security, and regulatory tools are one of the cornerstones of safety and quality.

## 8 Challenges and Integrated Solutions in Fish By‐Product Utilization

**FIGURE 4:**
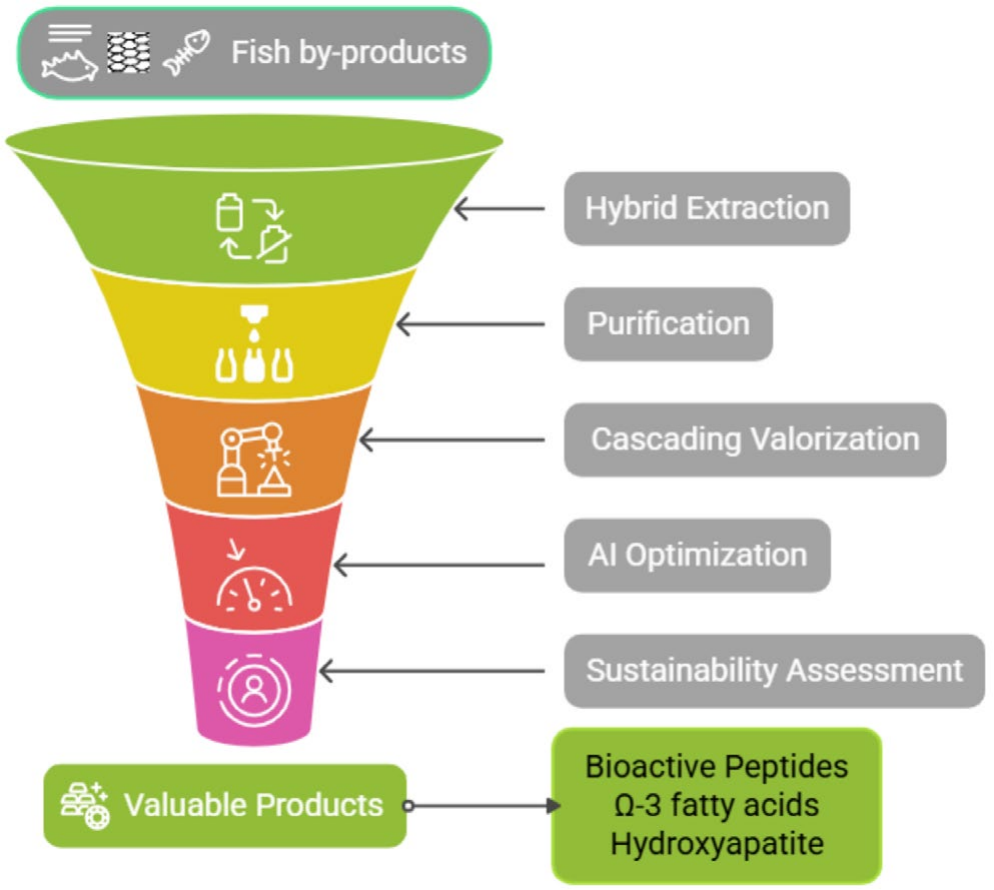
Integrated cascade valorization framework for fish by‐products.

The invention of fish by‐products, including skins, bones, heads, and scales, and viscera for the efficiency of obtaining valuable compounds such as collagen, peptides, and omega‐3s, may be a perfect solution in creating added value on fish, but there are moments of obstacles. These include technical variations, economic factors, effects on the environment, lack of legislation and regulations, and safety issues (Table 7). This section discusses these challenges and reviews advanced methods for integrating all the aspects of a bioeconomy for maximum optimization and less wastage in line with the circular economy principles to be adopted by biorefineries. This integrated approach, as illustrated in Figure 4, emphasizes a multi‐stage strategy for maximizing resource recovery and sustainability in fish by‐product biorefineries.

**TABLE 7:** Challenges and proposed solutions.

Category	Challenge	Impact	Proposed solution	Technological basis	References
Technical	Variability in by‐product composition	Inconsistent yields and bioactivity across species and seasons	Develop standardized protocols for different species	Enzyme‐assisted hydrolysis, AI‐driven optimization	Kurnianto et al. (2024)
Technical	Low yield of advanced extraction methods	Limited scalability of green technologies	Integrate hybrid methods combining enzymatic hydrolysis with ultrasound/pressure.	Ultrasound‐assisted extraction, supercritical CO_2_	Fahmy and El‐Deeb (2023)
Technical	Degradation during processing	Loss of bioactivity due to heat or chemical treatments	Use non‐thermal techniques such as cold plasma and subcritical water extraction	Cold plasma, subcritical water	Blondeau (2016)
Technical	Scalability of extraction methods	Difficulty in scaling up laboratory methods for industrial applications	Optimize process parameters using computational modeling	Aspen Plus software, machine learning	Kurnianto et al. (2024)
Economic	High initial investment	Cost barriers for small‐scale industries	Adopt biorefinery approaches to recover multiple products	Cascading valorization, integrated processes	Melgosa et al. (2020)
Economic	The low market value of traditional by‐products	Limited profitability compared to bioactive molecules	Increase value through advanced extraction and purification	Supercritical fluid extraction, nanofiltration	Alavi and Ciftci (2023)
Economic	Market competition	Competition with synthetic alternatives	Highlight sustainability and health benefits to attract eco‐conscious consumers.	Marketing strategies, consumer education	Yu et al. (2020)
Environmental	Disposal of extraction effluents	Environmental pollution from organic solvents and waste residues	Repurpose effluents for energy production (e.g., biomethane)	Anaerobic digestion, zero‐waste strategies	Ranade and Bhandari (2014)
Environmental	Lack of life cycle assessment (LCA)	Uncertainty regarding the ecological footprint	Conduct LCAs to optimize sustainability	Life cycle assessment tools	Melgosa et al. (2020)
Regulatory	Lack of global standards	Uncertainty about the safety and efficacy of bioactive molecules	Establish standardized protocols for extraction, purification, and quality control	Regulatory frameworks, third‐party certifications	He et al. (2019)
Regulatory	Heavy metal contamination	Potential toxicity in final products	Implement rigorous testing and purification processes	Allergen testing, encapsulation techniques	He et al. (2019)
Safety and quality	Stability and bioavailability issues	Reduced shelf life and efficacy of sensitive compounds	Use encapsulation techniques such as liposomes and nanoparticles	Nanotechnology, encapsulation	Zhang et al. (2019)
Safety and quality	Potential allergenicity	Risk of allergic reactions in sensitive populations	Conduct allergenicity assessments and develop hypoallergenic formulations	Bioinformatics, in vivo testing	Korkmaz and Tokur (2022)

### 8.1 Technical Challenges and Innovations

There are variations in the composition and structure of fish by‐products; for instance, skin protein presence ranges from 8% to 35% in different species. This requires specific approaches, hence making them even more complicated and expensive. For instance, enzymatic hydrolysis provides high‐quality peptides of cod skin but has some difficulties in the case of scale collagen because of the density of proteins (Korkmaz and Tokur 2022). Hybrid extraction also involves the use of enzymes in conjunction with UAE and results in an increased yield of 20%; furthermore, the process of extraction adapts to the change (Fahmy and El‐Deeb 2023). It should be noted that ML helps in estimating the best parameters, such as pH and the ratio of the enzymes, to result in a highly accurate estimate as compared to a guessed or tried and tested one (Kurnianto et al. 2024). Implementing a standard of operation that includes AI‐derived analytics would therefore promote species harmonization so that outputs are the same.

Nutrients such as omega‐3 fatty acids and peptides are prone to heat; this makes them degrade when exposed to heat. The process of hydrolysis affects the protein tertiary structure, leading to altered solubility (Ishak et al. 2021), while the process of gelatin extraction through heating leads to a similar impact on the yield of proteins (Silva et al. 2014). For instance, omega‐3 goes through oxidation during storage, leading to the reduction of the nutrients available to the body (Blondeau 2016). Green technologies like supercritical fluid extraction (SFE) and cold plasma preserve bioactivity. SFE recovers omega‐3s with minimal degradation (Ciriminna et al. 2017), and cold plasma enhances peptide antioxidant capacity by 15% via structural modifications (Hussain et al. 2020). Non‐thermal high‐pressure processing (HPP) doubles gelatin yield from bones compared to thermal methods, maintaining functionality. While enzymatic methods excel specifically, their scalability lags behind SFE, which offers higher purity but demands energy optimization. Hybrid systems (e.g., UAE + HPP) balance yield and integrity, but pilot‐scale validation is needed to confirm industrial viability.

### 8.2 Economic Barriers and Strategies

SFE systems alone are capital‐intensive and cost over 500,000$ (Rubio‐Rodríguez et al. 2010), hence a major hindrance for Scale industries as well. For instance, chemical hydrolysis yields fish meal, which is valued at 1–2 $/kg while collagen bioactive is priced at 9–14$/kg (Silva et al. 2014), and hence high processing costs reduce the profits. Biorefinery has adopted the concept of second generation to its models that have to do with cascading processing. Sequential extraction optimizes the removal of lipids through SFE, proteins by hydrolysis, and biogas by ADOC, with the advantage of minimizing waste output and costs (Melgosa et al. 2020). For instance, by‐product biorefineries of tuna bring omega‐3s and hydrolysates, resulting in increased revenue by 30%. Thus, it is noteworthy that PPPs can help subsidize infrastructure, given the examples of omega‐3 scale‐ups (Ciriminna et al. 2017). Integrated biorefineries are economically more effective than single‐output chains, but it is necessary to find suitable source feedstock like fish species in particular regions, such as Europe, which is abundant in cod. Though cost/benefits studies indicate that if scaled, hybrid methods could cut expenses by up to a quarter, the start‐up funding is still a fleeting element for developing countries.

### 8.3 Environmental and Regulatory Challenges

Solvent‐based extraction creates effluents that are dangerous to water and soil (Ciriminna et al. 2017). It also promotes the usage of the landfill, which becomes a hub of approximately 20 million tons of fish waste annually. Zero‐waste management recycles all effluents to generate biomethane in the form of methane through the process of anaerobic digestion, producing 200–300 m^3^ methane per ton of biomass, as explained (Ranade and Bhandari 2014). Subcritical water extraction reduces the usage of chemicals, which in turn reduces the emission levels by about 15% as opposed to chemical procedures (Hemker et al. 2020). LCAs help to minimize the environmental impact of the processes‐ SFE has a low carbon footprint for scaling that may take the firm to a new level (Blondeau 2016). Lack or absence of standardization of the safety measures of fish‐derived bioactive compounds fosters market risk and volatility. The risks of heavy contamination of metals, for instance, mercury in viscera, and allergenicity are well documented (Korkmaz and Tokur 2022), whereas the important controls of these issues are not established internationally (Ramakrishnan et al. 2024). Standardized guidelines, modeled on the EU Novel Food Regulation, could ensure safety. Nanotechnology‐aided purification (e.g., nanofiltration) removes 95% of contaminants like cadmium (Alavi and Ciftci 2023). Collaborative frameworks involving regulators and industry can harmonize testing, as piloted for collagen in cosmetics (Felician et al. 2018). Subcritical water extraction presents scalability challenges since it operates by minimizing environmental damage, while its water consumption must be improved. The implementation of regulatory standards remains slow, but the temporary acceptance of regional standards such as FDA GRAS would boost market readiness.

### 8.4 Safety and Quality Assurance

Heavy metals in fish waste and allergenic peptides from hydrolysis pose safety threats (Korkmaz and Tokur 2022). The stability of omega‐3 fatty acids improves by 40% with liposomal encapsulation, according to Zhang et al. (2019). At the same time, nanofiltration technology can separate non‐allergenic peptides from the mixture. Omega‐3 rancidity shortens the shelf life (Blondeau 2016). Testing costs decrease because ML screening identifies allergenic substances (Kurnianto et al. 2024). Heavy metal assays performed periodically throughout extraction aid in meeting the Codex Alimentarius maximum allowable levels. The absorption characteristics of peptides differ (bonito ACE inhibitors need human trial validation according to Korkmaz and Tokur (2022)) because pharmaceutical use remains restricted. Scientists have demonstrated that nanoparticle delivery platforms increase the amount of peptide absorption in the body by 30% (Alavi and Ciftci 2023). Research studies involving animal subjects should analyze peptide omega‐3 uptake to validate their therapeutic potential, just like krill oil. Encapsulating substances improve stability but add to manufacturing costs that need to be thoroughly analyzed. The lack of in vivo testing creates a critical research need because it would allow the merging of basic research with clinical data to open new therapeutic market opportunities, despite requiring significant funding.

## 9 Emerging Trends and Future Directions

The advancement of fish by‐product valorization requires integration between green technologies and interdisciplinary tools with sustainable practices. The subsequent part specifies emerging trends together with research directions to enhance Q1‐level impact despite current barriers.

### 9.1 Technological Frontiers

The application of cold plasma alongside subcritical water extraction produces sustainable processes that remove the need for solvents. The application of cold plasma increases peptide antioxidant activity levels by 15% while subcritical water extraction produces 10% more collagen output than chemical methods, yet requires no solvents (Hussain et al. 2020). The energy optimization required for scaling has HPP as a demonstration with its 50% energy reduction potential. Nanofiltration performs molecular weight‐based peptide fractionation, which supports specific applications (Bourseau et al. 2009). Nanoparticles act as selective extraction agents that extract 90% of omega‐3s from fish viscera according to Alavi and Ciftci (2023). Research into peptide bioactivity uses ML algorithms that achieve 85% accuracy in scientific discovery processes, so it speeds up the identification process (Kurnianto et al. 2024). Mathematical models have enhanced UAE process operations, which led to a 20% decrease in operating costs (Zhang et al. 2019). The combination of bioinformatics and metabolomics would build structure–function links to help create specific extraction methods, while nanotechnology delivers precise results yet demands the development of demonstration plants that define industry‐scale implementations. The potential of using AI for predictions for bioactive compounds is untapped to the extent that including more minor or obscure species may help scientists discover new elements.

### 9.2 Sustainable Valorization Strategies

The combination of cascading processes extracts lipids, peptides, and biogas, which reduces the amount of waste to lower than 5% (Melgosa et al. 2020). The production of omega‐3s and chitosan from krill biorefineries represents the implementation of Sustainable Development Goal 12. Biorefineries constructed in specific regions for invasive lionfish management provide solutions to environmental and economic concerns. The anaerobic digestion process of residuals leads to renewable energy production, which can save extraction costs by an estimated 10%. Sustainable operation depends on LCA analysis, which shows how SFE maintains lower emissions than standard practices according to Blondeau (2016). Biorefineries can create maximum value during production, but need customized optimization for each species. Global adoption of zero‐waste practices requires additional incentives, even though it already fits well with policy frameworks such as EU Blue Growth.

### 9.3 Research Priorities

Tests of peptide compounds for in vitro bioactivity (e.g., 68% DPPH scavenging) do not include human experimental data. Scientific research on bonito peptides should apply their proven antihypertensive potential from laboratory settings to clinical trials (Korkmaz and Tokur 2022). Additionally, scientists should investigate new biological resources such as Antarctic krill and unused fish byproducts, including sturgeon muscle. Evidence shows that phospholipid omega‐3s in krill achieve 30% better absorption than fish oil omega‐3s. The implementation of bioinformatics with nanotechnology and LCAs offers the potential to optimize holistic processes. ML‐guided nanofiltration integrates artificial intelligence for antihypertensive peptide extraction and lowers production expenses by 15% (Kurnianto et al. 2024). When prioritizing invasive species studies, researchers gain two advantageous outcomes related to ecological research objectives.

## 10 Conclusion

Fish by‐products previously perceived as waste in the seafood industry are now becoming invaluable sources of bioactive compounds that have enormous potential in food, nutraceutical, pharmaceutical, cosmetic, and agricultural industries. The diverse array of compositions of fish processing wastes, including those obtained from skins, bones, viscera, heads, scales, fins and frames, is highlighted in this extensive review as a source of rich sources of proteins (especially collagen and gelatin), essential fatty acids (e.g., omega‐3s), minerals (including hydroxyapatite and calcium carbonate) and bioactive peptides. These ingredients have a broad range of functionalities, such as antioxidants, antihypertensives, antimicrobials, antidiabetics, and anti‐inflammatories, and could prove to be more sustainable alternatives to synthetic additives and animal‐based ingredients.

Sophisticated extraction processes, especially enzyme‐mediated hydrolysis, membrane‐based fractionation (i.e., ultrafiltration and electrodialysis), and green‐based processing technologies like supercritical CO_2_ and subcritical water extraction have been of great use in enhancing the yield, purity, and bioactivity of derived molecules. Hybrid methods with ultrasound or pressure‐assisted processes, as well as enzymatic membrane reactors, provide additional opportunities to intensify processes and scale them. Ultrafiltration membrane electrodialysis is highly selective in collecting bioactive peptides according to charge and molecular weight, so that selective compounds with certain health‐promoting properties can be recovered. Furthermore, encapsulation technologies are developing innovations that are meeting important challenges associated with stability, bioavailability, and allergenicity, which enhance the shelf‐life and efficacy of finished products.

Although these improvements have been made, there are some issues that impede the complete valorization of fish by‐products. Technical factors like variability in the composition of the raw materials, degradation of the bioactive in the processing cycle, and the inability to scale up and translate laboratory procedures are also major obstacles. The costs of high initial investment and competition with the low‐value traditional uses or synthetic analogs will hamper market penetration, particularly among the small‐scale producers. Environmental issues such as effluent control and the absence of life cycle evaluations demand unified biorefinery designs that would respond to the ideas of the circular economy. Harmonization of regulations is also a pressing issue that must be undertaken to guarantee the safety of products, to standardize the quality control measures, and to create international market entry.

In the future, clinical validation of bioactive compounds needs to be a key priority of the research to justify health claims and provide an evidence base on which the health compounds will be approved by the health authorities. Standardized protocols that can be optimized using AI‐based modeling and real‐time monitoring systems are urgently needed and species‐specific. Moreover, the shift to zero‐waste systems in which each part of by‐products is used, and the unused streams are recycled to produce energy by an anaerobic digestion process, will be important to environmental sustainability. Transparency and market acceptance can be improved with the help of the integration of digital tools, blockchain traceability, and campaigns to educate consumers. Finally, the full potential of fish by‐products can be unlocked through interdisciplinary synergies between the scientists, industry stakeholders, and policymakers to grow a sustainable blue bioeconomy that turns waste into wealth and promotes functional nutrition and planetary health.

## Author Contributions

Muhammad Waqar: conceptualization (lead), methodology (lead), visualization (lead), writing – original draft (equal). Nimra Sajjad: methodology (equal), writing – original draft (equal). Faiyaz Ahmed: methodology (equal), writing – original draft (equal). S.S. Vasanthkumar and Temesgen Anjulo Ageru: visualization (supporting), methodology (equal), writing – original draft (equal). Qudrat Ullah: methodology (equal), writing – original draft (equal). Worawan Panpipat, Rotimi E. Aluko, Lovedeep Kaur, and Manat Chaijan: conceptualization (lead), investigation (lead), methodology (equal), project administration (lead), supervision (lead), validation (lead), writing – review and editing (lead).

## Ethics Statement

This study did not involve humans or animals.

## Consent

This study did not involve humans.

## Conflicts of Interest

The authors declare no conflicts of interest.

## Data Availability

The data supporting this study's findings are available from the corresponding author upon reasonable request.
